# Dietary Niches and Their Consequences for Five Insectivorous Passerines in a Seasonal Boreal Setting

**DOI:** 10.1111/mec.70620

**Published:** 2026-11-05

**Authors:** Coen Westerduin, Emma Vatka, Léo Lou Mael Chapuis, Oona Herzog, Seppo Rytkönen, Marko Mutanen, Markku Orell

**Affiliations:** ^1^ Ecology and Genetics Research Unit University of Oulu Oulu Finland; ^2^ Department of Environmental and Biological Sciences University of Eastern Finland Joensuu Finland; ^3^ Ecological Genetics Research Unit University of Helsinki Helsinki Finland; ^4^ École Normale Supérieure PSL University Paris France

**Keywords:** climate change, diets, food webs, Lepidoptera, metabarcoding, passerines

## Abstract

To understand the effects of climate change and other disturbances on ecosystems, methodologies capable of unravelling the complexity of biotic interactions are vital. Here, we present a dietary study on five passerines nesting in boreal forests: the crested tit *Lophophanes cristatus* , willow tit *Poecile montanus* , blue tit *Cyanistes caeruleus* , great tit *Parus major* , and pied flycatcher *Ficedula hypoleuca* . We used metabarcoding to describe their arthropod prey during the breeding season, and linked diets' properties with breeding success to understand birds' dependence on particular prey. The birds' diets contained hundreds of arthropod taxa, showing considerable richness and interannual variation. Most prey identities were resolved to species level. The most common prey were Lepidoptera, followed by Diptera, Coleoptera and Araneae. Dietary overlap was low to middling, conformed to temporal overlap, and fluctuated across the three studied years. Higher consumption of lepidopterans and their larvae was strongly associated with higher breeding success in blue tits and, to a lesser extent, great tits. In contrast, crested tits, willow tits and pied flycatchers had a more constant breeding success without relying on specific prey. The results suggest that the more recently settled blue tits and great tits are less capable of optimally utilizing the comparatively resource‐poor boreal environments. The apparent good adaptation to local foraging conditions contrasts to declining population trends in the Red‐Listed crested tits and willow tits. Our study highlights the complexity of arthropod communities and insectivore food webs, and showcase how molecular methods can be used to study ecological interactions.

## 1 Introduction

From intense agriculture, forestry and urbanization to global climatic change, anthropogenic actions are directly and indirectly reshaping the natural world. For some species, these highly variable environmental changes have accommodated expansions beyond their historical ranges, with populations increasing in the process (Hällfors et al. 2021). Other species have seen their habitats fragment and shrink, leading to population declines and local or even global extinctions. The same organism might well experience both processes in different parts of their range, resulting in an overall shift in distribution. Similarly, as seasonal patterns change with increasing temperatures, organisms may shift their timing of seasonal behaviours (Burger et al. 2012).

A wide range of physiological and genetic factors will determine whether and to what extend organisms can respond and sufficiently adapt to changing environmental conditions, both short‐term and at evolutionary timescales. In addition, both the need for and ease of adaptation are impacted by biotic interactions (Visser et al. 1998). However, it has long been difficult, if not impossible, to practically detect and map interactions involving large numbers of species, hampering our understanding of the potential cascade of shifting interactions across the complex networks in whole ecosystems. Recent advances in especially molecular techniques are now allowing ecologists to start unravelling such hitherto cryptic interactions (e.g., Shutt et al. 2020).

Here, we present a dietary study involving five bird species and their arthropod prey, conducted over 3 years in Finnish boreal forests during the birds' nesting season. The five small passerines represent most local members of the family Paridae (crested tit *Lophophanes cristatus* (Linnaeus, 1758), willow tit *Poecile montanus* (Conrad von Baldenstein, 1827), blue tit *Cyanistes caeruleus* (Linnaeus, 1758), great tit *Parus major* , Linnaeus, 1758) and one belonging to Muscicapidae (pied flycatcher *Ficedula hypoleuca* (Pallas, 1764)). Finnish crested tit and willow tit populations have drastically declined in recent decades, with both now Red‐Listed (Hyvärinen et al. 2019). In contrast, blue tit and great tit populations have expanded their ranges northward over the last century. The past decades have also seen substantial, but variable, changes in the distributions and seasonal timings of local lepidopterans (Hällfors et al. 2021; Yazdanian et al. 2023), whose larvae especially are considered important prey for these birds (Both et al. 2009; Rytkönen et al. 1996; Rytkönen and Orell 2001; Van Balen 1973). As phenologies of the lepidopterans advance with increasing spring temperatures, the focal birds relying on these as prey for their nestlings are expected to correspondingly shift their timings of breeding. If these shifts occur at different rates, it may create a costly mismatch for the birds (Both and Visser 2001; Visser et al. 1998). Yet, the rates of shifts and their levels of synchrony have been found to be far from universal in this system, with substantial intraspecific and interspecific variation (Bailey et al. 2022; Burger et al. 2012; Eeva et al. 2012; Valtonen et al. 2017; Vatka et al. 2011, 2014; Visser et al. 2003). While ongoing climatic warming is particularly strong at higher latitudes (Trenberth and Hurrell 2019), thus far limited phenological shifts or evidence for mismatches have been observed in this context (Bailey et al. 2022; Vatka et al. 2014). A key, but thus far cryptic, factor may lie in differences in the arthropod communities, and the birds' differential utilizations of these.

To elucidate this facet, this study has two main objectives:

1. To describe and explain the interspecific and intraspecific variation in the arthropod taxa that make up the nestling diets of the five bird species in a boreal setting.
2. To investigate the fitness consequences of those diets on the different bird species.

These goals are achieved by way of extracting taxonomic information from arthropod DNA in the nestlings' faeces using metabarcoding (1), and then linking these dietary data to the breeding data from the same nests to investigate the fitness consequences of those diets (2).

For objective (1), we hypothesize that a combination of different associations with specific prey resulting from the birds' historical distributions, differences in the exact breeding times, and different foraging techniques utilizing different tree species or parts thereof (Rytkönen and Krams 2003) will lead to a limited dietary niche overlap among these birds. We expect that birds that are breeding comparatively early (crested tit) or late (pied flycatcher), and have a longer history of breeding in the comparatively resource‐poor boreal forests (crested tit, willow tit, and pied flycatcher), will have more diverse diets with low quantities of any particular prey. Given the expected importance of phenological synchrony in this system, we further expect diets of birds breeding around the same time (willow tit, blue tit, and great tit) to feature more of the same prey, even if at different quantities due to differing foraging strategies.

Contrasting to the aforementioned population trends, individual nests of both blue tits and great tits often perform worse than those of other parids in our boreal setting (Mägi et al. 2009; Rytkönen and Orell 2001; Rytkönen and Krams 2003). As such, we expect for objective (2) that optimal preferred foraging conditions will be more impactful for these species, with higher proportions of Lepidoptera in the diets associated with larger broods of nestlings in better condition. A weaker or no effect is expected for the other species, predicting these to be better adapted to local conditions.

By looking into an often overlooked or simplified part underlying the phenological tree–insect–bird study system, we aim to find if niche differentiation can help explain the previously described variation in phenological shifts. This information will help in understanding current and predicting future impacts of climate change on the natural world.

## 2 Material and Methods

### 2.1 Field Area, Nest Monitoring, and Sampling

The focal birds have been studied in the boreal forests near Oulu, Finland (approx. 65°05′ N 25°33′ E) as part of long‐term monitoring for several decades (Orell et al. 1999). Samples and breeding data used here were collected in 2018–2020. During this time, the study area covered some 30 km^2^, 350 nest boxes and 1000 provided rotten stumps—the former the primary nesting place for blue tits, great tits, and pied flycatchers; the latter for crested tits and willow tits. The area contains predominantly Scots pine ( *Pinus sylvestris* ), Norway spruce ( *Picea abies* ), and birch (*Betula* spp.), but has heterogeneity resulting from intermittent swamps, and variations in the density and ages of these trees due to forestry.

Nests were regularly visited (generally weekly, but as often as daily when monitoring hatching) to track fundamental breeding data, including clutch size, nestling survival, and parental identities. Nestlings were routinely individually ringed and measured (licence 180 from the Finnish Ringing Centre) aged 13 days (willow tits and blue tits) or 14 days (crested tits and great tits), during which faecal samples were also collected: nestlings commonly defecate when handled (King et al. 2008). This age reflected the period of peak energetic needs for the nestlings (Royama 1966). Nests without living nestlings at this stage could not be sampled and are thus not part of this study. Otherwise, nestling mortality was counted both when it occurred between hatching and ringing or between ringing and fledging. Fresh faecal sacs were collected from bird bags, weighing containers, or the researchers' hands or clothing, but to limit severe environmental contamination not from the ground. Faeces were collected in ethanol‐filled 5 mL tubes, pooled per brood, and deposited in a freezer at −20°C later on the day of collection. While not ringed, pied flycatcher broods were also sampled aged 13 days by transferring the nestlings to a bird bag and container before returning them to the nest minutes later, which generally yielded faeces from most handled nestlings.

Monitoring and ringing measurements provided the data for our fitness metrics: mean nestling weight, number of fledglings, and probability to fledge (based on the number of hatched eggs and number of fledglings). Well‐developing nestlings should be approaching their maximum weights at the measured age (Orell 1983), providing information on their condition as fledglings. The open population, near‐continuous forest cover extending beyond the study area, and limited sampling years precluded the use of, for example, recruitment data as a viable measurement. Identities and ages of the parents were obtained during ringing of the parents trapped at their nests. Age categories of ‘yearling’ (i.e., born in the preceding year) or ‘older’ (2+ years old) were assigned to parental birds based on either tail feather condition and shape (crested tits and willow tits) or the colour of covert feathers (blue tits and great tits; L. Svensson 1992). Alternatively, we knew the ages for individuals ringed by us as nestlings or breeding parents in previous years, and categorized them accordingly. Pied flycatcher adults were neither ringed nor aged.

### 2.2 Sample Selection, Preparation and Processing

To balance representation across species and given practical constraints, we selected a subset of the collected samples for metabarcoding, chosen from across the breeding periods and area, including additional samples from the peak of each bird species' breeding period. We removed samples likely belonging to second broods (based on repeated parental identities or late breeding times), as neither their diets nor fitness would be typical of the main breeding period. Breeding times of the sampled birds are illustrated in Figure S1. From here onwards, Figures and Tables will list species as: *L. cri* = crested tit; *P. mon* = willow tit; *C. cae* = blue tit; *P. maj* = great tit; *F. hyp* = pied flycatcher; in the same (breeding period) order.

Laboratory procedures and bioinformatics largely followed a protocol by Rytkönen et al. (2019) with the updates from Westerduin et al. (2023); for brevity we will only summarize the main steps and record any deviations here. Subsamples of 250 mg (or as much as was available) were taken from homogenized faecal samples, extracting DNA utilizing QiaAmp Fast DNA Stool Mini Kit (Qiagen). We amplified ~150 bp of the arthropod mtDNA from the standard COI barcode region with ZBJ‐ArtF1c/ZBJ‐ArtR2c primers (Zeale et al. 2011), capable of high amplification and species discrimination in the main taxon of interest, Lepidoptera (Clarke et al. 2014). PCR amplifications took place in two steps (amplifying the target sequence region in the first, and adding sample‐specific tags and adapters in the second); the first step was performed in duplo for each sample (subsequently merging them) to counteract inherent stochasticity, with success checked via gel electrophoresis. Sequencing was performed on the Ion Torrent PGM (Thermofisher) at the Biocenter Oulu (University of Oulu), which also provided basic bioinformatics (demultiplexing, denoising, chimera removal). Samples were processed and analysed in batches of 150 (which included samples outside of the scope of this study), mostly in the year following collection. Negative controls were only sequenced in later batches due to new insights in the sensitivity to contamination (Westerduin et al. 2023); prior to that they were kept until gel electrophoresis only, where they gave no visible signal. Samples displaying no viable DNA based on gels were excluded from sequencing.

Table 1 gives the final sample numbers; limited samples for the crested tit mainly resulted from a smaller local breeding population—we otherwise balanced sample selection across species.

**TABLE 1:** Sample numbers (successfully sequenced) used in this study, listed by bird species for each breeding season.

Year	Bird species				
	*L. cri*	*P. mon*	*C. cae*	*P. maj*	*F. hyp*
2018	7	28	28	27	25
2019	11	27	25	23	30
2020	19	26	20	20	20
Total	37	81	73	70	75

Since sampling covered several years, shared nesting locations and/or parental identities were present in subsequent years. We nonetheless expected diets and samples to be suitably independent due to the highly dynamic nature of arthropod communities. We tested this assumption by separately comparing all samples from nests for which the site and both parents were fully identical across years. Identical sites were defined as either the exact same nest box (for blue tits and great tits) or a generally newly excavated nesting cavity within 100 m of a previous year's nest (for crested tits and willow tits).

### 2.3 Taxonomic Assignments

Per batch, all Amplicon Sequence Variants (ASVs) were first ran in BOLD's (boldsystems.org; Ratnasingham and Hebert 2007) ‘Species Level Barcode Records’ Identification Engine, and subsequently queried using the less detailed ‘All Barcode Records’ data if no match could be found in the former. Finnish arthropods, especially Lepidoptera, are particularly well‐represented in this database (Roslin et al. 2021). If several equally close matches were given, we favoured locally‐occurring taxa (laji.fi; gbif.org)—given sparse records on many arthropods besides Lepidoptera, we considered occurrences anywhere in the country to be sufficient, whereas lepidopterans required occurrences within tens of kilometres. A handful of taxa at least described for neighbouring countries were accepted if displaying unambiguous good matches and no local alternatives were available; this included new species first described in recent years, plausibly present but undescribed locally. When several equally probable matches remained, the assigned identity was the lowest shared taxonomic level. We used a 98% threshold for matches (King et al. 2015), but given our short sequences, decided to also accept matches 97%–98% on the condition that the same taxon was found elsewhere in the data at 98% match or higher. This was aimed at preventing losing data to minor sequencing errors or incompleteness of reference data for all haplotypic variants: even a > 97% match accepts just 4 mismatched nucleotides in the short barcodes. ASVs lacking identities were assigned taxonomic information using alternative methods (see below) if passing subsequent filters.

### 2.4 Database Construction and Missing Data

Quantitative filtering of the metabarcoding data followed Drake et al. (2022), combining relative per‐sample thresholds with absolute thresholds informed by negative controls. Given our few sequenced negative controls, we chose a conservative 0.5% relative read abundance (RRA; Deagle et al. 2019), and 2× max. reads found for any ASV in any negative control. Abundances of remaining ASVs were normalized to 100% per sample, with RRA used as the quantitative metric in analyses. Retained ASVs lacking identities were ran in BOLD an additional time in early 2024 to consider new reference material added since the first identifications were conducted (in 2019). ASVs still missing identities were subsequently queried in Protax (https://laji.fi/en/theme/protax; Roslin et al. 2021; Somervuo et al. 2016), at 140 bp overlap and probability threshold 0.5, adding the most detailed provided taxonomic information to the existing data. Synonyms were resolved to the standard name used by the Finnish Biodiversity Information Facility (FinBIF). ASVs still lacking assigned identities were kept with unique sequentially numbered NM (‘no match’) identifiers. Finally, abundances for each unique identity across the data were merged.

To the fitness data, we similarly added unique sequential identifiers for any unobserved or unringed female parents and assigned them as age ‘unknown’. This included all pied flycatchers as well as two willow tits, two blue tits, and eight great tits. Other missing data (caused by missing observations or defective measuring equipment) were assigned NA; NA rows were excluded in their relevant analyses.

### 2.5 Supplementary Data on Common Lepidopterans

Additional ecological and other biological traits for abundant lepidopterans were collected from literature—predominantly Silvonen et al. (2014), I. Svensson (1993), and laji.fi. These encompassed defence strategies (urticating setae, mining, rolling/folding leaves (hereafter: ‘rolling’), webbing, casing, or free‐living), habitat parameters (canopy‐dwelling, (near‐)obligatory birch specialist (hereafter: ‘birch’), deciduous and/or coniferous trees as hosts), and available life stages (egg, larva, pupa, and/or imago). The life stages were defined as their availability to the birds during three time windows: late May–early June, mid‐June, late June–early July. These roughly correspond to the sampling periods of respectively crested tits; willow tits, blue tits, and great tits; and pied flycatchers. ‘Abundant lepidopterans’ was defined as species occurring in ≥ 10 samples overall and/or having an average RRA > 1% in the diet of at least one bird species. We added whether pupae were realistically accessible to the focal birds. For larval‐specific analyses, we included any species potentially available as larvae—but possibly also other life stages—during a time window, not merely those exclusively available as larvae. Larval life stages usually co‐occurred with pupae (of which most were inaccessible to the birds, though accessible specimens are plausibly widely consumed; Van Balen 1973) or less commonly eggs (which we are not aware of being fed to nestlings, even if accessible).

For non‐parametric multivariate analyses, only accessible life stages (on a per‐prey species basis) were included, and consumed prey were summed per sample for each possible life stage separately. While cumulative proportions of prey consumed as eggs + larvae + pupae + imagines per sample could thus exceed 1, this would help reveal patterns of any favoured life stages more clearly than to split proportions over multiple simultaneously available life stages (of which some might not be consumed). Other uncertainties were implemented similarly, counting species as having multiple defence strategies even if a specific larva would not employ them simultaneously (e.g., *Parornix anglicella*, where young larvae mine and older ones roll up leaves). We summed prey with certain defence strategies or habitats only for prey possibly available as larvae in the bird species' relevant time window (per the aforementioned criteria) since these predominantly described larval qualities.

### 2.6 Analyses and Illustrations

Where relevant, results were first calculated as averages per bird species per year, and then averaged across years, to give equal weight to each year.

We calculated dietary overlap among the birds with Pianka's niche overlap index (Pianka 1973), separately for all prey, all lepidopteran prey, and all lepidopteran larval prey (see above). The last of these only covered the mid‐period breeding birds (willow tits, blue tits, and great tits), as only these were fully comparable by encountering prey in the same life stages. Overlap was judged to be biologically significant if > 0.6 (Grossman 1986).

Resemblance matrices were calculated and analysed in Primer‐E 6.1.13 (Clarke and Gorley 2006) with Permanova+1.0.3 (Anderson et al. 2008). We conducted permutational multivariate analyses of variance (PERMANOVA, at 999 permutations) to explore similarities between the birds' diets. Distance‐based linear models (DistLM) were fitted to analyse the relevance of prey traits to the birds' diets. Data distributions were illustrated with principal coordinate (PCO) plots. In addition to the aforementioned lepidopteran prey traits, DistLM analyses included as possible predictor variables: year, May date (continuous day counts starting from the 1st of May), latitude (roughly corresponding to distance from urbanized areas in the study sites), and sequencing run. Post hoc pairwise DistLM analyses were conducted for each combination of bird species. Best fitting models were selected based on the lowest corrected Akaike information criterion (AICc; Burnham and Anderson 2002) and Bayesian information criterion (BIC; Schwarz 1978) scores for any combination of included variables. AICc was selected for the comparatively many explanatory variables compared to sample sizes (well below the 1:40 ratio threshold; Burnham and Anderson 2002), while stricter BIC scores highlight the most relevant variables. DistLM analyses for higher taxonomic levels only included the host plant and sample‐related variables, excluding species‐specific prey defence or life stage traits.

We analysed fitness consequences of the diets in R 4.3.2/4.3.3 (R Core Team 2023), using packages lme4 (Bates et al. 2015), Rcpp (Eddelbuettel and François 2011), effects (Fox 2003), and ggplot2 (Wickham 2016). Normality of residuals was checked visually with Q‐Q plots (commands ‘qqplot’, ‘qqline’) prior to later steps. We ran nine models covering all combinations of three fitness response variables *y* (mean nestling weight, number of fledglings, probability to fledge) and three diets (proportion of lepidopteran larvae, proportion of Lepidoptera, Levins' niche breadth; Levins 1968). This model has as a basic structure:

(1) $$y=\beta_{1}\text{bird species}+\beta_{2}\text{female}\;\mathrm{age}+\beta_{3}\text{bird species}\times \text{diet}+\text{female identity}+\text{year}+e$$

where *β*s are regression coefficients for fixed term effects, female identity and year are block random variables, and *e* a residual term. Intercept term and diet as an independent factor were purposefully excluded from the model structure so that the effects would bind into the ‘bird species’ and ‘bird species × diet’ terms, respectively. This model structure allows for the most direct comparison of the effect of the diets on the different bird species. Female identity was omitted from ‘number of fledglings’ models due to singular fit of the models when included. Nestling weight models used linear mixed‐effect models (function ‘lmer’ in lme4). ‘Number of fledglings’ or ‘probability to fledge’ (latter included as a two‐column variable of *N*(fledged young) (1 s) and *N*(non‐fledged young) (0 s)) used generalized linear mixed models (‘glmer’ in lme4), with Poisson and binomial distributions respectively. The probability model required a switch of the optimizer term to ‘bobyqa’ with maximum number of iterations of 100,000. Subsequent pairwise bird species comparisons followed the same structure (Equation 1), but included a separate non‐interaction ‘diet’ fixed factor, and each bird species separately used as a baseline. Degrees of freedom (df) were calculated as:

(2) $$\mathrm{df}=N\left(\text{observations}\right)-N\left(\text{fixed effect estimates}\right)-N\left(\text{random effect levels}\right)$$

Species accumulation plots were created using the package BiodiversityR (functions ‘accumcomp’, ‘accumcomp.long’; Kindt and Coe 2005) at default settings, giving the expected (mean) species richness and standard deviations for a given number of samples following 100 permutations.

Statistical significance was based on *α* = 0.05, though full *p*‐values are presented wherever appropriate for more nuanced interpretations.

## 3 Results

### 3.1 Samples and Their Contents

We selected 369 faecal samples for DNA extraction, of which 26 were discarded based on gel electrophoreses. Failed samples occurred across years and species but disproportionally for crested tits, which typically had small samples owing to smaller broods. The average numbers of faecal sacs per sample were: crested tit 2.4, willow tit 3.2, blue tit 4.2, great tit 3.4, and pied flycatcher 4.5. The highest total read numbers in a negative control were 36; highest reads for one ASV 7. Upon removing one crested tit sample containing few reads following quantitative filtering steps (max. 19 for any ASV), a final 336 samples remained (Table 1), excluding negative controls. The average (±SD) number of reads in the final bird samples were: crested tit 9567 (±8373), willow tit 12,565 (±9169), blue tit 11,459 (±4415), great tit 11,830 (±9109), pied flycatcher 12,852 (±4856).

Initial taxonomic assignments could be made based on 100% matches to BOLD's reference data for 56% of ASVs, and 5% were assigned identities based on 97%–98% matches. 76% of the identities were assigned to species level, 10% to genus, and 4% to family (frequently dipterans). We found no acceptable match for 8% of ASVs at this stage. Re‐querying using BOLD added identities to 9 ASVs; Protax identified 56 of the 79 remaining ASVs: none to species level, 18 to genus, 14 to family, and the rest higher taxonomic levels. Most of the 27 unique identities assigned via Protax covered taxa already present in the data (albeit with representatives resolved to lower taxonomic levels); two taxa were wholly new (Hippoboscidae and Sphaeroceridae).

After merging read counts for ASVs with identical identities, our data featured 532 unique prey, henceforth referenced as ‘prey species’—in actuality, identities were resolved to species level for 429 of these, 68 to genus, 19 to family, and no match for 14. No‐match ASVs accounted for 0.22% of reads (SE 0.07%) in the samples on average; excluding those, samples contained an average of 11.47 (SE 0.29) distinct prey species.

### 3.2 Dietary Descriptions

Lepidoptera were the most abundant of the 17 encountered arthropod prey orders, at 56%–92% across the birds, followed by Diptera (Table 2, Figure 1). Average dipteran consumption exceeded 20% for great tits and pied flycatchers. Noteworthy lesser abundances were from Coleoptera (7% for crested tits, 10% for pied flycatchers) and Araneae (5% for crested tits). Interannual variation was apparent (Figure S2), with Lepidoptera generally more prominent in 2018 compared to 2019 and especially 2020; relative abundances of Lepidoptera were consistently the highest for blue tits (> 89%).

**TABLE 2:** Prey orders in the birds' diets (relative read abundances, means 2018–2020). No value listed if fully absent.

Prey orders	Bird species				
	*L. cri*	*P. mon*	*C. cae*	*P. maj*	*F. hyp*
Lepidoptera	70%	75%	92%	75%	56%
Diptera	12%	17%	6%	21%	27%
Coleoptera	7%	1%	1%	2%	10%
Araneae	5%	1%	< 1%	1%	< 1%
Hymenoptera	< 1%	2%	< 1%	1%	1%
Hemiptera	1%	2%	< 1%	< 1%	< 1%
Siphonaptera	2%	< 1%		< 1%	1%
Blattodea	< 1%	< 1%		< 1%	2%
Psocodea	2%		< 1%	< 1%	< 1%
Megaloptera	< 1%		< 1%		1%
Neuroptera	< 1%	< 1%		< 1%	< 1%
Sarcoptiformes	< 1%	< 1%		< 1%	< 1%
Trombidiformes		< 1%	< 1%	< 1%	
Trichoptera		< 1%			< 1%
Odonata					< 1%
Orthoptera				< 1%	
Opiliones					< 1%

**FIGURE 1:**
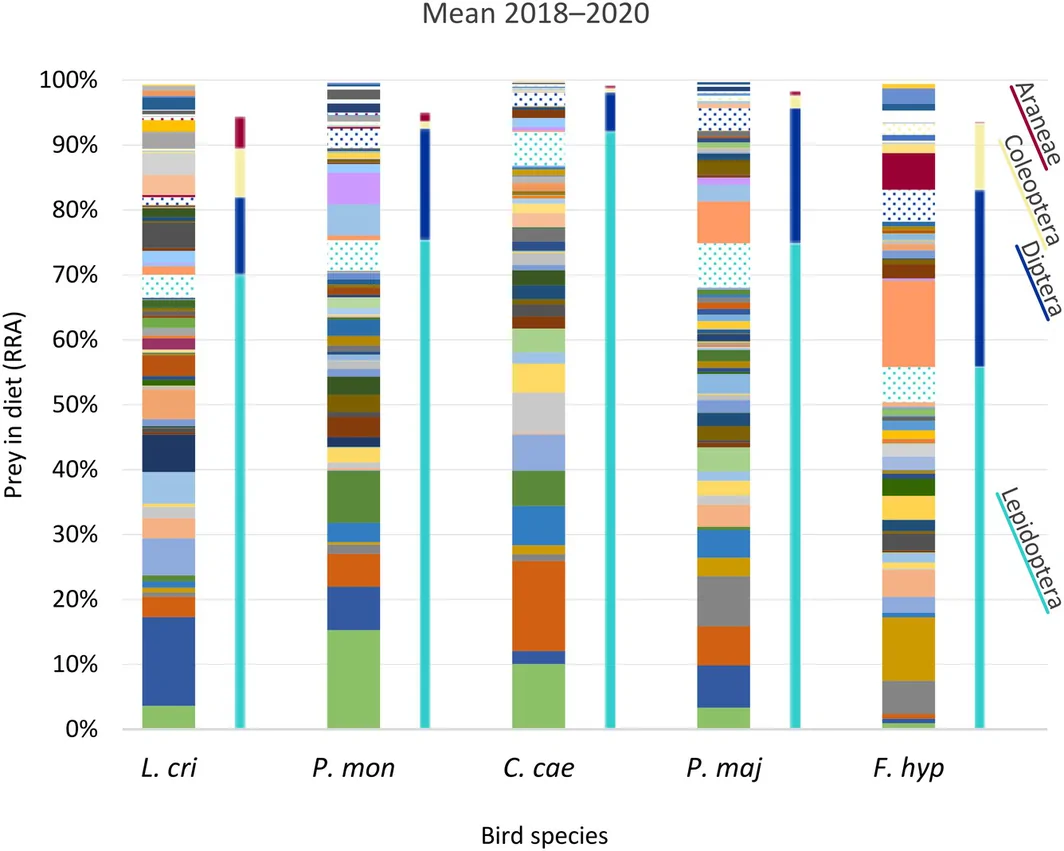
Mean diets expressed as relative read abundances (RRA). The main bars display the 100 most abundant prey overall, grouped by order, in descending order of overall abundance. Patterned bar segments indicate sums of remaining species in prominent orders. Corresponding orders are shown as narrow bars to their right. Legend omitted due to the excessive length; full details in Table S1.

Only a handful of the hundreds of prey species had abundances above a few percent (Figure 1, Table S1). Of 12 prey species with average abundances > 5% in any diet, ten were lepidopterans ( *Epirrita autumnata* , *Hyppa rectilinea*, *Operophtera brumata* , *Bupalus piniarius*, *Epinotia crenana*, *Hydriomena furcata* , *Geometra papilionaria*, *Panolis flammea*, *Brachylomia viminalis*, *Hylaea fasciaria*), one a dipteran ( *Rhagio scolopaceus* ), and one a coleopteran (*Pterostichus oblongopunctatus*). Species abundant in one diet were often found in several and sometimes all the birds' diets, and frequently across several—but not necessarily all—years. For instance, *B. piniarius* was a common prey for several birds in 2019 and 2020, yet absent in 2018. The samples from repeated pairs of parents from comparable nesting sites displayed limited dietary similarities between years: any notable shared prey were typically common across the diets in a given year for the bird species in question (Figure S3). We thus decided that the samples from different years could be considered (sufficiently) independent, even though recurring parental birds and breeding areas presented risks of pseudoreplication.

The 79 most abundant lepidopteran species included for life stage‐specific analyses represented 89%–96% of consumed lepidopterans for the bird species. Relative to the full diet, few larvae were generally detected in the crested tit diets, consistently many in the blue tit's, and moderate values otherwise (Table 3). The lower values were not merely due to lower proportions of lepidopterans in the diets of some birds (Figure 1); larvae also made up a small proportion of overall Lepidoptera eaten by crested tits, whereas for pied flycatchers most of the comparatively few lepidopterans eaten were larvae.

**TABLE 3:** Yearly and average consumption of lepidopteran larvae (relative read abundances, mean ± SE). Lepidopteran prey were considered to have been consumed as larvae if available as larvae during the relevant time window.

Year	Bird species				
	*L. cri*	*P. mon*	*C. cae*	*P. maj*	*F. hyp*
2018	43% ± 12%	71% ± 5%	82% ± 3%	48% ± 6%	40% ± 4%
2019	14% ± 7%	28% ± 5%	75% ± 6%	29% ± 5%	44% ± 6%
2020	22% ± 8%	46% ± 6%	79% ± 4%	46% ± 6%	61% ± 6%
Average	26%	48%	79%	41%	48%

Dietary richness was highest for the pied flycatcher, followed by the great tit; taking into account sample numbers, observed richness appeared similar in the crested tit, willow tit, and blue tit (Figure 2). Slopes for the great tit and especially pied flycatcher remained increasing when approaching the maximum number of samples available. Absolute as well as relative patterns varied across years (Figure S4). Where pied flycatchers and great tits typically had high dietary richness, crested tits were in line with great tits in 2019 but scored lower than any other birds in 2020 despite comparably high sample numbers. Plateauing was less clear across birds in individual years.

**FIGURE 2:**
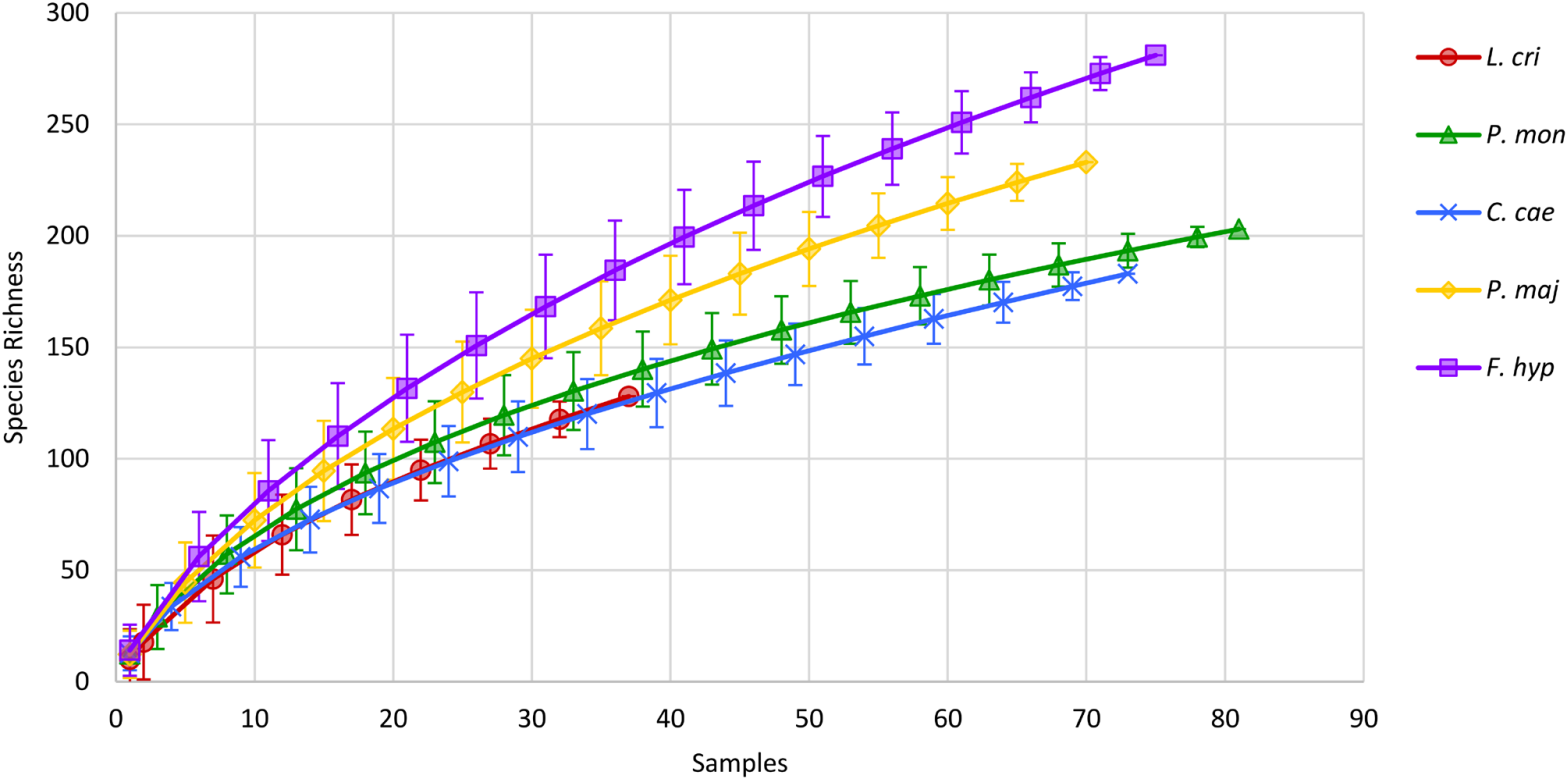
Species accumulation curve for all dietary samples. Error bars display 2 × SD. Only the first, last, and otherwise every fifth sample were plotted (at different intervals for the different bird species) for improved discernability. For individual years, see Figure S4.

### 3.3 Niche Overlap

Similarities for the full diets were low–medium among the birds (Figure 3). Pied flycatchers had the most distinctive diets, only showing higher overlap with great tits; most overlap was seen among the willow tit, blue tit, and great tit. Most species' diets showed less overlap in 2019 than 2018, and even lower overlap in 2020 (Figure S5). Most dramatically, overlap between crested tits and great tits ranged from 2018's highest score (0.69) to the (shared) lowest in 2020 (0.10). Comparatively high overlap between the willow tit and blue tit was more stable (0.49–0.55). When separately analysing the overlap only for consumed lepidopterans, scores were generally marginally higher than those for the full diet (Figure 3, Figure S4). When only considering the lepidopterans likely consumed as larvae, we found higher overlap still (Figure 4). Mean scores exceeded 0.6 for both the willow tit × blue tit and blue tit × great tit comparisons. The highest value across the data (0.87) was found for the willow tit × blue tit overlap for lepidopteran larvae in 2019 (Figure S6).

**FIGURE 3:**
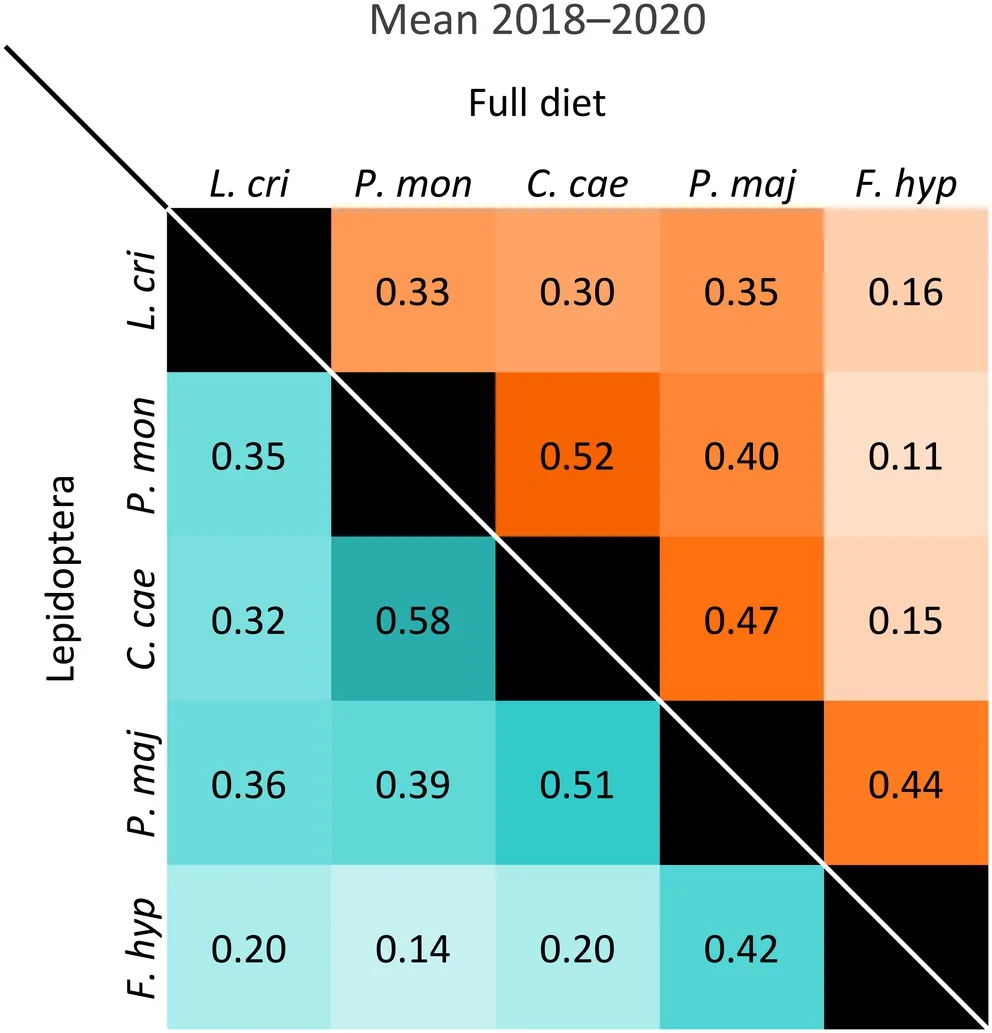
Pianka's niche overlap scores averaged over the sampled years. The top right shows the overall overlap for the full diets, the lower left shows scores for the Lepidoptera in the diets only; darker colours indicate higher overlap in either. For individual years, see Figure S5.

**FIGURE 4:**
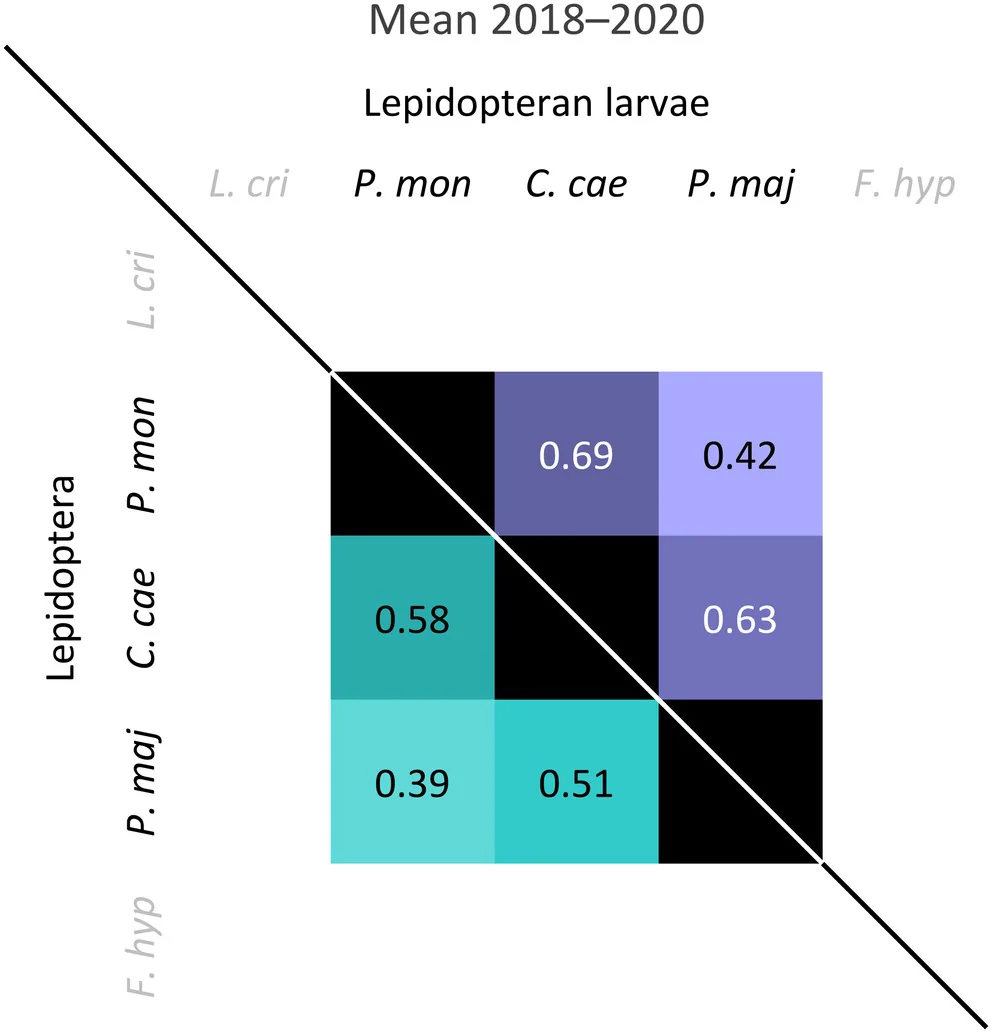
Pianka's niche overlap scores averaged over the sampled years. The lower left shows the overlap for the Lepidoptera in the diets, the top right shows the scores for the lepidopteran larvae only; darker colours indicate higher overlap in either. White numbers indicate values > 0.6. For individual years, see Figure S6.

Further analyses found almost all birds to be clearly distinct (PERMANOVA; crested tit × pied flycatcher *t* = 1.135, *p* = 0.126, willow tit × blue tit *t* = 1.287, *p* = 0.029, all other pairwise comparisons *t* ≥ 1.329, *p* < 0.005). Crested tits showed especially low similarity within the species, while it was high between willow tits and blue tits (Table 4). Little variation in the overall data distribution could be explained by any PCO axes (Figure 5, Table S2). Although all prey trait and sample variables had statistically significant effects, each explained only little of the variation in the data (Table S3). Overall, models excluding latitude, sequencing run, and/or year scored marginally better than those including all variables (AICc; Table S4). The association of consumed prey with deciduous trees was the single most impactful metric (Tables S3 and S4). A combination of some prey defence strategies (prominently leaf rolling), habitat data (with birch not necessarily aligned with overall deciduous trees; Figure 5), life stage data, and May date was the most informative (AICc and BIC, Table S4). Pairwise comparisons among the birds highlighted the same variables, with only urticating setae, year, latitude, and sequencing run not included in any of the best models (BIC, Figure 6, Table S5). At higher taxonomic levels, the explanatory power of the PCOs increased considerably (Figures S7 and S8) and models became simpler (Tables S6 and S7). However, the patterns differentiating species were less clear (Figures S7 and S8, Tables S8 and S9), and the statistical significance and explanatory power of some variables dropped considerably (Figures S7 and S8, Tables S10 and S11). Prey associations with deciduous or coniferous trees and May date remained the most important factors explaining differences among the birds' diets (Tables S6, S7, S10 and S11).

**TABLE 4:** Average similarities between and within bird species, following PERMANOVA with 999 permutations, S17 Bray–Curtis similarity.

	*L. cri*	*P. mon*	*C. cae*	*P. maj*	*F. hyp*
*L. cri*	5.304				
*P. mon*	5.087	12.491			
*C. cae*	5.540	11.197	15.687		
*P. maj*	4.339	6.529	7.527	7.276	
*F. hyp*	3.166	2.711	3.084	5.993	12.793

**FIGURE 5:**
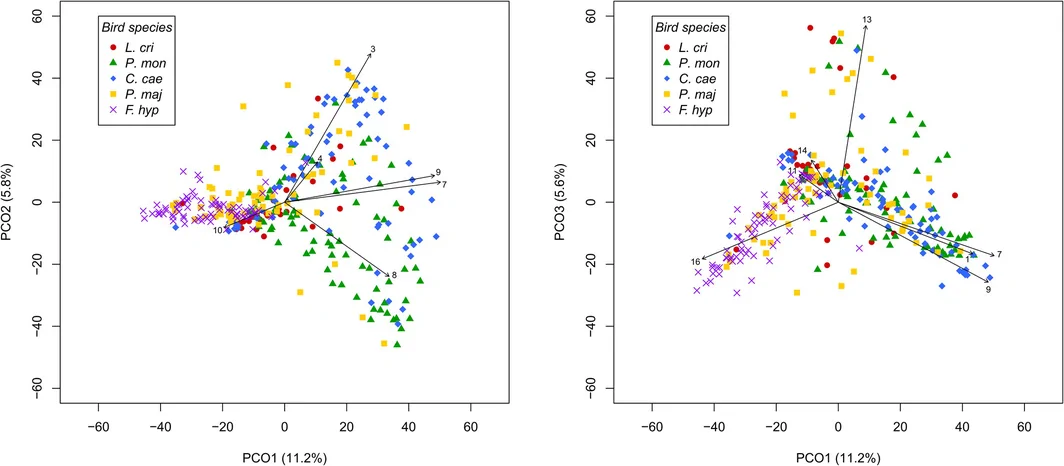
PCO ordinations of the bird species' diets. Percentages next to the PCO axis titles refer to the percentage of variation explained by each. Vectors display Pearson correlations of prey traits and other variables, with relative lengths corresponding to their correlations to the axes. Only variables included in the best fitting DistLM models (lowest BIC and scores within 1) are shown (Table S4), plotted if correlating to the relevant axes > 0.5, or with whichever pair of PCO axes the correlation was highest to. Variables: 1 = Free‐living, 3 = Rolling, 4 = Webbing, 7 = Canopy, 8 = Birch, 9 = Deciduous, 10 = Coniferous, 11 = Egg, 13 = Pupa, 14 = Imago, 16 = May date.

**FIGURE 6:**
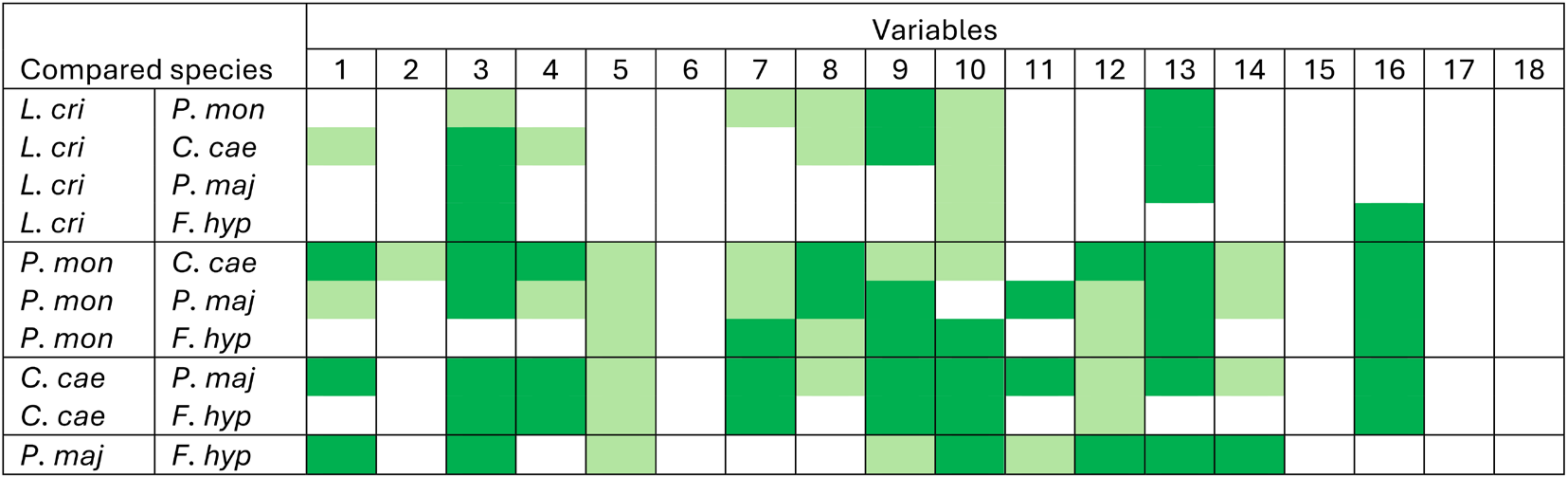
Best DistLM solutions (lowest BIC and scores within 1) for pairwise comparisons of the bird species. Variables included in the best model shown as dark green; variables in other models with scores within 1 shown as lighter green. Note that not all variables would have been included in the same combinations; see Table S5 for full details. Variables: 1 = Free‐living, 2 = Mining, 3 = Rolling, 4 = Webbing, 5 = Casing, 6 = Urticating setae, 7 = Canopy, 8 = Birch, 9 = Deciduous, 10 = Coniferous, 11 = Egg, 12 = Larva, 13 = Pupa, 14 = Imago, 15 = Year, 16 = May date, 17 = Sequencing run, 18 = Latitude.

### 3.4 Diets' Impacts on Fitness

Increased consumption of lepidopteran larvae and lepidopterans in general was positively associated with nestling weight in blue tits (Figure 7, Table S12). Numbers of fledglings were less clearly associated with our dietary metrics; they did increase with female age (Figure 7, Table S13). The probability of a hatchling to fledge was strongly positively associated with lepidopterans in the diet for blue tits, and for both blue tits and great tits when only considering lepidopteran larvae (Figure 7, Table S14). Pairwise comparisons of the birds showed significant differences between blue tits and all other species beside great tits for the same models (Tables S12–S14). Levins' niche breadth showed no clear impacts on any fitness metrics (Tables S12–S14), but blue tits and great tits differed significantly for this metric alone in the ‘probability to fledge’ model, with blue tits showing a more negative association (Figure 7, Table S14). Although annual variation was apparent throughout analyses, any overall impact of year on the fitness metrics was weak (Table S15).

**FIGURE 7:**
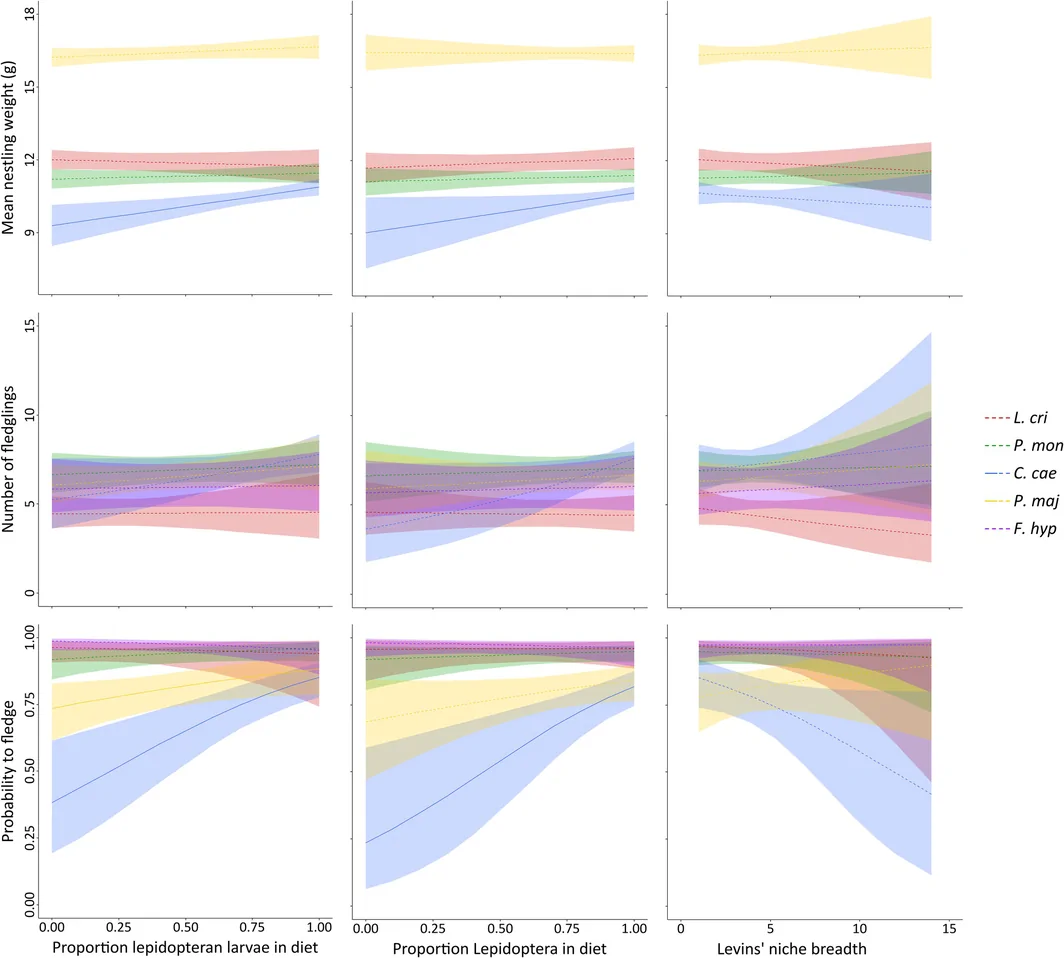
Effects of dietary metrics on fitness variables. Semi‐transparent ribbons display 95% confidence limits. Solid lines represent effects for *bird species* × *diet* (Equation 1) with *p*‐values < 0.05; interrupted lines are given if *p* > 0.05. For full details, see Tables S12–S14.

## 4 Discussion

In this study, we investigated the arthropod prey relevant to five passerines during their breeding seasons. Our first objective was to describe and compare the birds' diets. These featured hundreds of prey species, and our results showcase the potential complexity of insectivore food webs resulting from the highly diverse arthropod communities. Substantial interannual dietary variation furthermore highlights the importance of multiannual studies in such contexts. We found generally moderate dietary overlap among the birds, with a handful of common prey shared among several or all bird species. Dietary overlap was more prominent among those with more overlap in their breeding periods (willow tits, blue tits, great tits), and most pronounced for lepidopteran larvae. Fine‐scale niche differences were hinted at by various models, with the taxonomic detail provided by metabarcoding foundational to finding these differences. Our second objective was to investigate the fitness consequences of those diets. The partially shared diets did not directly translate to shared reliance on similar prey. Specifically, a lower proportion of lepidopteran larvae in the diets corresponded to fitness costs only in blue tits and, less severely, great tits.

### 4.1 Dietary Descriptions

This study is the first to provide detailed accounts of the prey of these birds in a boreal context, with high accuracy and depth enabled by the extensive COI reference data available for Finnish arthropods, especially lepidopterans (Roslin et al. 2021). The comprehensiveness of the reference library also reduced the risk of mistaking pseudogenes (NUMTs) for species; a risk for short barcode sequences (Hebert et al. 2023). Existing dietary information on these birds was generally restricted to broad prey categorizations (but see Shutt et al. 2020; Verkuil et al. 2022) and from central or southern European contexts (e.g., Atiénzar et al. 2009; Michalski et al. 2011; Naef‐Daenzer and Nager 1998; Nour et al. 1998; Pagani‐Núñez et al. 2011; Van Balen 1973). We found taxonomically detailed information invaluable in describing differences within and between the bird species. Few of the hundreds of prey taxa were abundant and many rare, reflecting the abundance distribution typical for arthropod communities (Simons et al. 2015). Some uncommon prey may however be valuable to the birds for specific compounds (e.g., carotenoids for pigmentation). The most consumed lepidopterans are (periodically) locally abundant species, largely arboreal with larvae feeding on Scots pine (*B. piniarius*, *P. flammea* , *H. fasciaria*) and deciduous trees ( *E. autumnata* , *O. brumata* , *H. furcata* , *G. papilionaria*, *B. viminalis* and *H. rectilinea*). The high dietary richness and variable diets between years paint a picture of highly opportunistic predators. Although our study was not designed to test these hypotheses directly, the variation within samples or between years does not suggest a strong influence of a potential ‘search image’ (see also: Royama 1970) or individual specialism (Pagani‐Núñez et al. 2016). Any benefits of these may locally be outweighed by interannual variation in prey availabilities and the comparatively low arthropod densities at higher latitudes (Vatka et al. 2011). That said, the dietary differences between bird species do also suggest levels of specialism within the lepidopteran‐focussed insectivores. The partially unique diets combined with potentially preferred prey shared among the birds highlight how insectivores' food sources are not necessarily interchangeable (Mittelbach and McGill 2019; Reeves et al. 2021).

Even when covering hundreds of species, our dietary descriptions should not be regarded as exhaustive even in this specific context. Methodological limitations in primer design preclude the equal amplification of all taxa while maintaining high taxonomic resolution across the board (Jusino et al. 2019). With our choice of primers, we here focussed on species coverage and taxonomic detail in Lepidoptera: preferred both by the more generalist and specialist focal bird species (Rytkönen et al. 1996; Rytkönen and Orell 2001). Lepidopterans and their larvae are rich in lipids for nestling development (Razeng and Watson 2015; Reeves et al. 2021) and central to the phenological mismatch context (Visser et al. 1998). In using these primers, we may have overlooked certain underamplified taxa (Clarke et al. 2014). For instance, we found few sawflies, although their larvae are expected to be relevant for some boreal parids (Vatka et al. 2016). That said, previous research could not distinguish these from lepidopteran larvae (Rytkönen et al. 1996), and they might not be preferred food owing to comparatively low nutritional value (Razeng and Watson 2015), making limited consumption plausible. Similarly, where pied flycatchers might have been expected to consume woodlice (Samplonius et al. 2016), we here found none. Our primers have previously successfully amplified terrestrial isopods however (Razgour et al. 2011). A genuine (near)‐absence in the local birds' diets may be a consequence of very low woodlouse abundances in central–northern Finland compared to the lower latitudes the pied flycatchers have previously largely been studied in. Primer cocktails can mitigate amplification biases and enable better taxonomic coverage (Corse et al. 2019; De Barba et al. 2014). However, combining the resulting data into a single coherent quantitative dataset can be problematic (da Silva et al. 2019), hampering subsequent statistical analyses (for the contentious topic of quantitative metabarcoding in general, see, e.g., Deagle et al. 2019; Verkuil et al. 2022). Moreover, we expect presence/absence data to especially strongly skew insectivore diets, overestimating the importance of small but frequently ingested arthropods (possibly including parasites) compared to larger prey of which few individuals will be present in any meal and dropping. We preferred not to remove any prey taxa based on their assumed likelihood as prey, thus possibly retaining parasites or arthropods consumed through secondary consumption (Sheppard et al. 2005; Verkuil et al. 2022). However, with DNA (and subsequently reads) from such sources expected to have comparatively low abundances, utilizing read numbers reduces their impact. Indeed, we here found relative read percentages of likely parasites to be low (e.g., Siphonaptera, Table 2). Importantly, the comparative nature of this study means that biases are shared between birds, and found dietary differences likely genuine. Crucially, we expect additional prey data to reinforce the patterns described herein: further increase dietary diversity, further reduce relative contributions of any particular prey, and create further differences between the birds' diets.

### 4.2 Niche Differences and Fitness Consequences

Matching our hypothesis, bird species with more overlap in breeding times often displayed more dietary overlap. High overlap between crested tits and great tits in 1 year may be a stochastic effect owing to a low sample size for the former, though high dietary overlap across that year suggests potentially high abundances of favoured lepidopterans to also have played a role. In other years, the early‐breeding crested tits are likely restricted by limited early‐season arthropod availability. Crested tits' dietary proportion of Lepidoptera was the second lowest, with few consumed as larvae (even using a wide definition of prey likely consumed as larvae). While the proportion of lepidopterans was the lowest in the diets of the late‐breeding pied flycatchers, compensated by high dipteran intake, most lepidopterans appeared to be eaten as larvae by them. Although consumed prey life stages are partially a factor of seasonal timing, the inclusion of various life stages alongside May date in best‐fitting models suggest different preferences or encounter rates in utilized (parts of the) trees to also play a role (Rytkönen and Krams 2003). Blue tits stood out as consistently having a lepidopteran larvae‐focussed diet—the proportions of Lepidoptera in the diets were lower and more variable among years in willow tits and great tits. This specialization indicated a reliance as per our fitness analyses, with blue tit nestlings heavier and more likely to fledge in nests for which consumption of lepidopteran larvae was high. Since we could not study the diets in broods without surviving nestlings at the time of sampling, our analyses were limited to at least somewhat successful nests, and the real impact could be stronger yet. Samples from single measurements are also not expected to be representative of broods' diets throughout the nestling phase: indeed, the diets are known to change corresponding to nestling development (Royama 1970; Samplonius et al. 2016; Van Balen 1973). With single, pooled samples from the time of peak energetic needs (Royama 1966), we aimed to balance relevance, sampling effort, DNA extraction success, affordability, and analytical simplicity. While pooled faecal samples per nest might have skewed dietary richness in larger broods, this was not evident from the blue tit's narrow diet—if anything, there was an association between diverse diets and low breeding success in this species. In contrast, we found no reliance on any particular prey in most of the other bird species. Whereas great tits also appeared to benefit from lepidopteran larvae in raising nestlings to fledge, they contrasted with blue tits in their more positive association with dietary diversity.

Part of the differences can be attributed to the ‘brood reduction’ breeding strategy (Orell 1983) which may more commonly be practised by local blue tits and great tits, captured in our fitness metric ‘probability to fledge’. Notably, we not only found no evidence for any fitness costs in crested tits, willow tits, or pied flycatchers, but this divergence existed despite reasonable dietary overlap among several of these bird species. Intriguingly, the reliance on lepidopteran larvae mirrors the birds' time since local settlement—a possible example of maladaptation following dispersal to unfamiliar areas (Nosil et al. 2005). Since the last glacial period, great tits and blue tits have shifted their distributions northwards from southern Europe (Kvist et al. 1999, 2004), expanding their breeding ranges to northern Finland as recently as the 1950s and 1980s respectively (Veistola et al. 1994 in: Kvist et al. 1999; Rytkönen and Krams 2003). Great tits have previously been considered to lay higher‐than‐optimal numbers of eggs at high latitudes (Rytkönen and Orell 2001). Our findings suggest blue tits and great tits may furthermore suffer from suboptimal foraging decisions there. The former's specialism poorly fits fluctuating, low‐resource boreal conditions (Veistola et al. 1997), while the latter may be physically or behaviourally limited in optimally utilizing available foraging substrates (Rytkönen and Krams 2003). While both are thought to focus their foraging efforts on deciduous trees, the other focal birds have been considered to associate more with conifers (Burger et al. 2012; Rytkönen and Krams 2003), and Estonian great tits have previously been described as breeding more successfully in less‐preferred coniferous forests (Sisask et al. 2010). Coniferous areas see a less pronounced and later larval peak (Mägi et al. 2009), which fits larval consumption patterns found for crested tits (low) and pied flycatchers (high). Still, many commonly consumed lepidopterans had deciduous host plants, and contrary to our expectations we found willow tits to be associated with those prey more than great tits were. Partially as a result, it was between willow tits and blue tits that we found the highest dietary similarities overall, despite their differing historical distributions. While all birds forage on all of the few abundant local trees to some extent, prey host plant was among the most pervasive factors differentiating the birds' diets. That said, we were only able to explain a fraction of dietary variation in our models, with diverse interactions and intricate foraging strategies plausibly inherent to insectivore food webs such as these. Lepidopteran larvae may be preferentially targeted by blue tits and great tits for their high carotenoid levels, with these brighter‐coloured species plausibly requiring more of the food‐derived pigments (Eeva et al. 2005). Even so, longer‐time resident crested tits and willow tits, and the long‐distance migrating pied flycatchers, appear better able to balance food requirements with local conditions. Interestingly, they share this ability despite limited niche overlap, indicating multiple viable strategies across the season and forest types.

The seemingly good adaptation contrasts to the crested tits' and willow tits' declining national population trends (Hyvärinen et al. 2019). However, willow tit declines may mainly be driven by factors outside of the breeding season (Lehikoinen et al. 2024). Competition with the more recently settled parid species has been suggested (Müller 2024), but countered for the willow tit at least (Lehikoinen et al. 2024). Given limited dietary overlap and almost fully separate breeding times, we consider it likely that the crested tit has too distinct a niche to be strongly impacted by such competition during the breeding season. The distinct but sometimes similar diets of the birds should be regarded in the context of generally highly diverse diets, and with shared prey (both within and between bird species) being locally abundant arthropods.

### 4.3 Implications for Ongoing Adaptation and Future Research

Over the last decades, several of the focal birds have been the subjects of intense study across their range in relation to variable advancing seasonal phenologies of themselves and their arthropod prey (e.g., Both and Visser 2001; Burger et al. 2012; Vatka et al. 2011, 2014; Visser et al. 1998). Bailey et al. (2022) hypothesized that phenological shifts at high latitudes have remained limited, despite strong climatic effects there, due to a lower dietary specialism compared to more prey‐dense central European areas. Our findings support this notion, describing highly diverse diets for boreal populations.

Our research highlights the complexity of the seasonal insect–insectivore study system. While these birds have long been known to have diverse diets even at lower latitudes (Betts 1955), for practical reasons studies tend to equate their prey to all lepidopterans (e.g., Both et al. 2009; Vatka et al. 2011; Visser et al. 1998). The birds' specific and variable diets can help explain the poor explanatory power of total frass as a proxy for prey availability (Mägi et al. 2009), and they are integral in understanding their different breeding periods and dissimilar phenological shifts. With even just lepidopterans already displaying highly variable phenological and distributional shifts in response to climatic change (Fält‐Nardmann et al. 2016; Hällfors et al. 2021; Valtonen et al. 2014; Yazdanian et al. 2023), improved knowledge of the whole food peak architecture (Vatka et al. 2016) across species, areas, and time is required to stand any chance of understanding both these dynamic food webs, and the complex impacts of climate change on the natural world in general.

## Author Contributions

Original study design by C.W., E.V., S.R., M.O.; sample collection by C.W., E.V., O.H., S.R., M.O.; extra data collected by M.M.; pilot studies by L.L.M.C., O.H., with input from C.W., E.V., S.R.; analyses by C.W., E.V.; manuscript lead author C.W. All authors contributed to the manuscript, and consented on its submission.

## Funding

This work was supported by Suomen Kulttuurirahasto, Technology and Natural Sciences Doctoral Programme at the University of Oulu, Research Council of Finland (Profi6 336449), Kvantum‐instituutti, Oulun Yliopisto and Koneen Säätiö (202103581, 202008514).

## Disclosure

Benefit‐sharing statement: All research including sample analyses took place in the country of sample collection, by researchers from multiple nationalities, largely permanently residing in this country. Any researchers and students with sufficient contributions were invited as authors. Relevant generated and compiled data has been made publicly available on Dryad as below. The research addresses topical and urgent issues of the impact of climate change on natural systems and provides ecological insights relevant to several Red‐Listed species.

## Ethics Statement

This research was performed in accordance with Finnish laws and regulations. Bird handling and ringing was conducted under permit nr 180 from the Finnish Ringing Centre.

## Conflicts of Interest

The authors declare no conflicts of interest.

## Supporting information


**Figure S1:** Breeding times of the sampled bird populations over three breeding seasons.
**Figure S2:** Mean yearly diets expressed as relative read abundances (RRA).
**Figure S3:** Diets of repeated parents, separated by species.
**Figure S4:** Species accumulation curves for the individual years.
**Figure S5:** Pianka's niche overlap scores for the sampled years.
**Figure S6:** Pianka's niche overlap scores for the sampled years.
**Figure S7:** PCO ordinations of the bird species' diets analysed by prey family.
**Figure S8:** PCO ordinations of the bird species' diets analysed by prey order.
**Table S1:** Overview of the 100 most abundant prey species (or lowest‐level identities available) in the birds' diets, in descending order (relative read abundances) of taxonomic orders, then descending order of abundance of species within each.
**Table S2:** Pearson correlations between the PCOs and the explanatory variables.
**Table S3:** Results of marginal tests for DistLM solutions for all prey species and sample variables.
**Table S4:** Best DistLM solutions for different numbers and combinations of variables, based on both lowest AICc and BIC scores.
**Table S5:** Best DistLM solutions (lowest BIC and scores within 1) for pairwise comparisons of the bird species.
**Table S6:** Best DistLM solutions (lowest AICc or BIC and scores within 1) for diets analysed by prey family.
**Table S7:** Best DistLM solutions (lowest AICc or BIC and scores within 1) for diets analysed by prey order.
**Table S8:** Average similarities between and within bird species, following PERMANOVA with 999 permutations, S17 Bray–Curtis similarity, for diets analysed by prey family.
**Table S9:** Average similarities between and within bird species, following PERMANOVA with 999 permutations, S17 Bray–Curtis similarity, for diets analysed by prey order.
**Table S10:** Results of marginal tests for DistLM solutions for all prey species and sample variables for diets analysed by prey family.
**Table S11:** Results of marginal tests for DistLM solutions for all prey species and sample variables for diets analysed by prey order.
**Table S12:** Results from models describing the effects of diet compositions on nestling weight in four bird species.
**Table S13:** Results from models describing the effects of diet compositions on number of fledglings in five bird species.
**Table S14:** Results from models describing the effects of diet compositions on the probability to fledge in five bird species.
**Table S15:** Results for random effects for the models describing the effects of diet compositions on the birds' fitness.

## Data Availability

Feature tables of raw and filtered metabarcoding data (including identities assigned to species and sample information), as well as compiled prey information, breeding parameters, and additional data on samples used for models are available on Dryad: https://doi.org/10.5061/dryad.8931zcs4j.
