# Supplementary material for: Dietary Niches and Their Consequences for Five Insectivorous Passerines in a Seasonal Boreal Setting
Source: Mol Ecol. 2026 Nov 5;35(21):e70620. doi: 10.1111/mec.70620 (PMC13735998; doi:10.1111/mec.70620)
Supplement: Supplementary file 1 [file MEC-35-e70620-s001.pdf]

MOLECULAR ECOLOGY

Supplemental Information for:

Dietary niches and their consequences for five insectivorous passerines in a seasonal boreal setting

Westerduin, Coen; Vatka, Emma; Chapuis, Léo; Herzog, Oona; Rytkönen, Seppo; Mutanen, Marko; Orell, Markku

Table of Contents:

**Figure S1**	Page 2
**Figure S2**	Page 3
**Table S1**	Page 5
**Figure S3**	Page 8
**Figure S4**	Page 10
**Figure S5**	Page 12
**Figure S6**	Page 13
**Table S2**	Page 14
**Table S3**	Page 15
**Table S4**	Page 16
**Table S5**	Page 17
**Figure S7**	Page 20
**Figure S8**	Page 21
**Table S6**	Page 22
**Table S7**	Page 22
**Table S8**	Page 22
**Table S9**	Page 22
**Table S10**	Page 23
**Table S11**	Page 23
**Table S12**	Page 24
**Table S13**	Page 26
**Table S14**	Page 28
**Table S15**	Page 30

MOLECULAR ECOLOGY

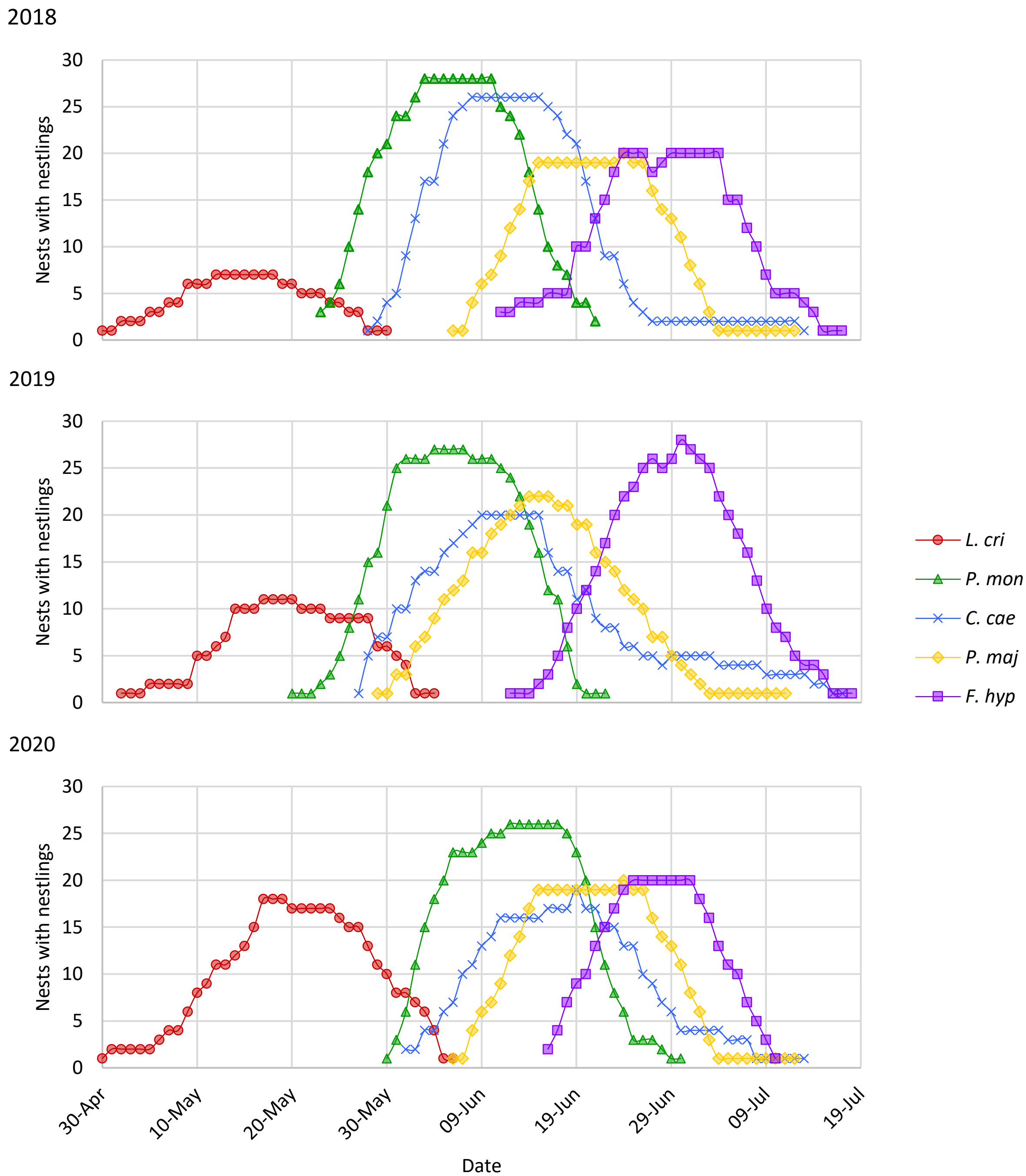

Figure S1. Breeding times of the sampled bird populations over three breeding seasons. Only sampled nests are included, from the moment the first nestlings of a brood hatched, up until their fledging date (for simplicity, here assumed to consistently be at the age of 15 days for *F. hyp* and 18 days for the other species).

MOLECULAR ECOLOGY

2018

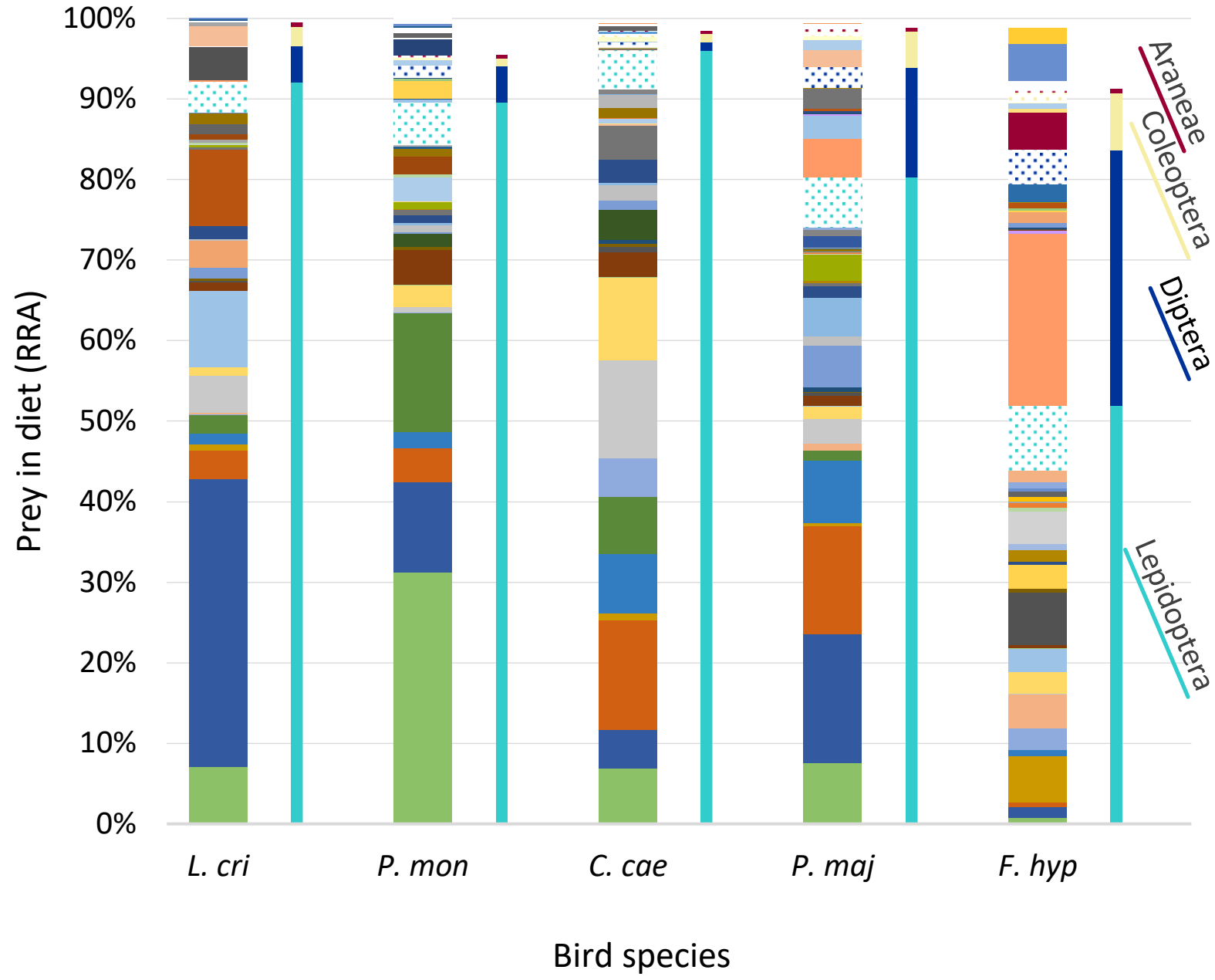

2019

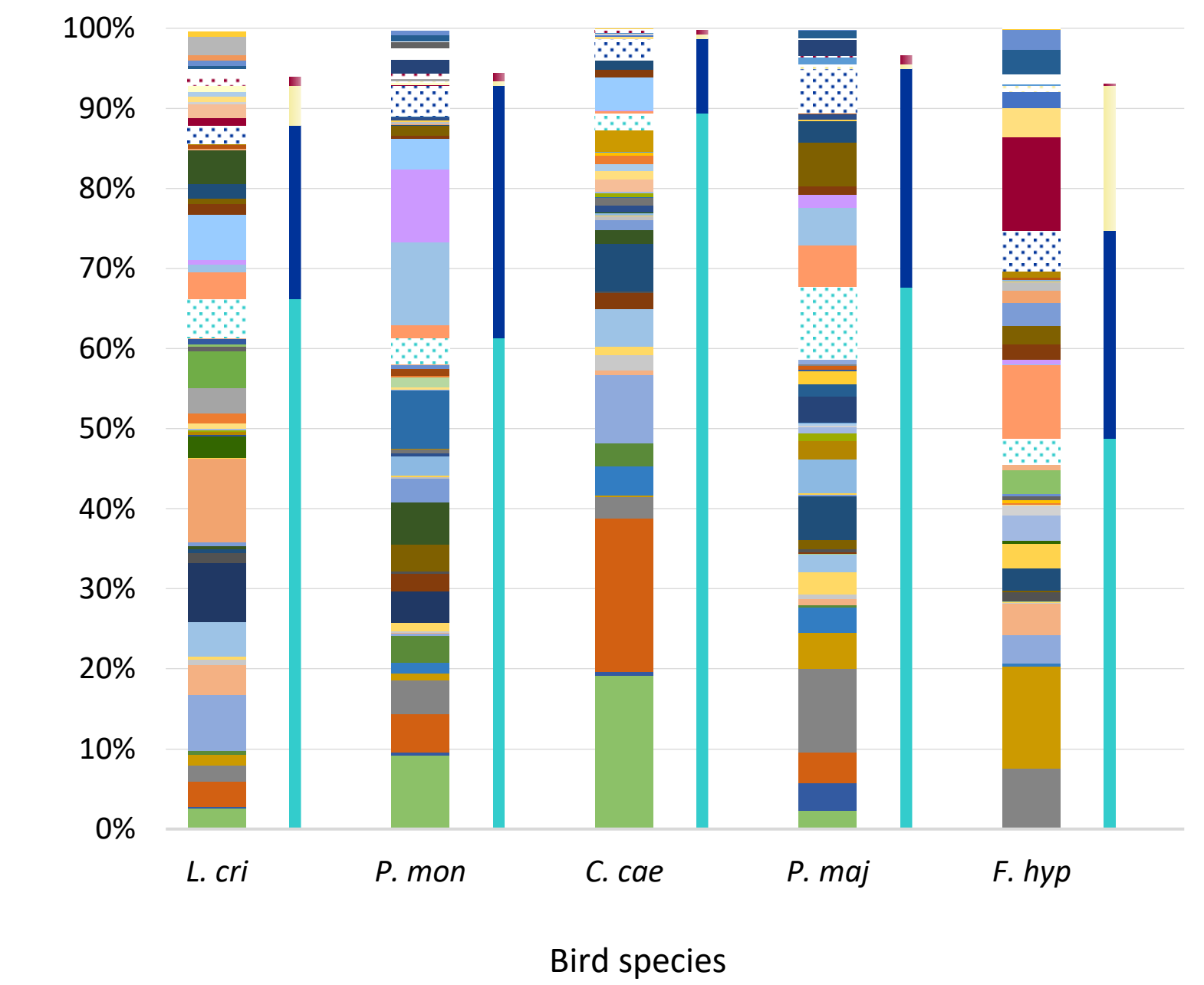

MOLECULAR ECOLOGY

2020

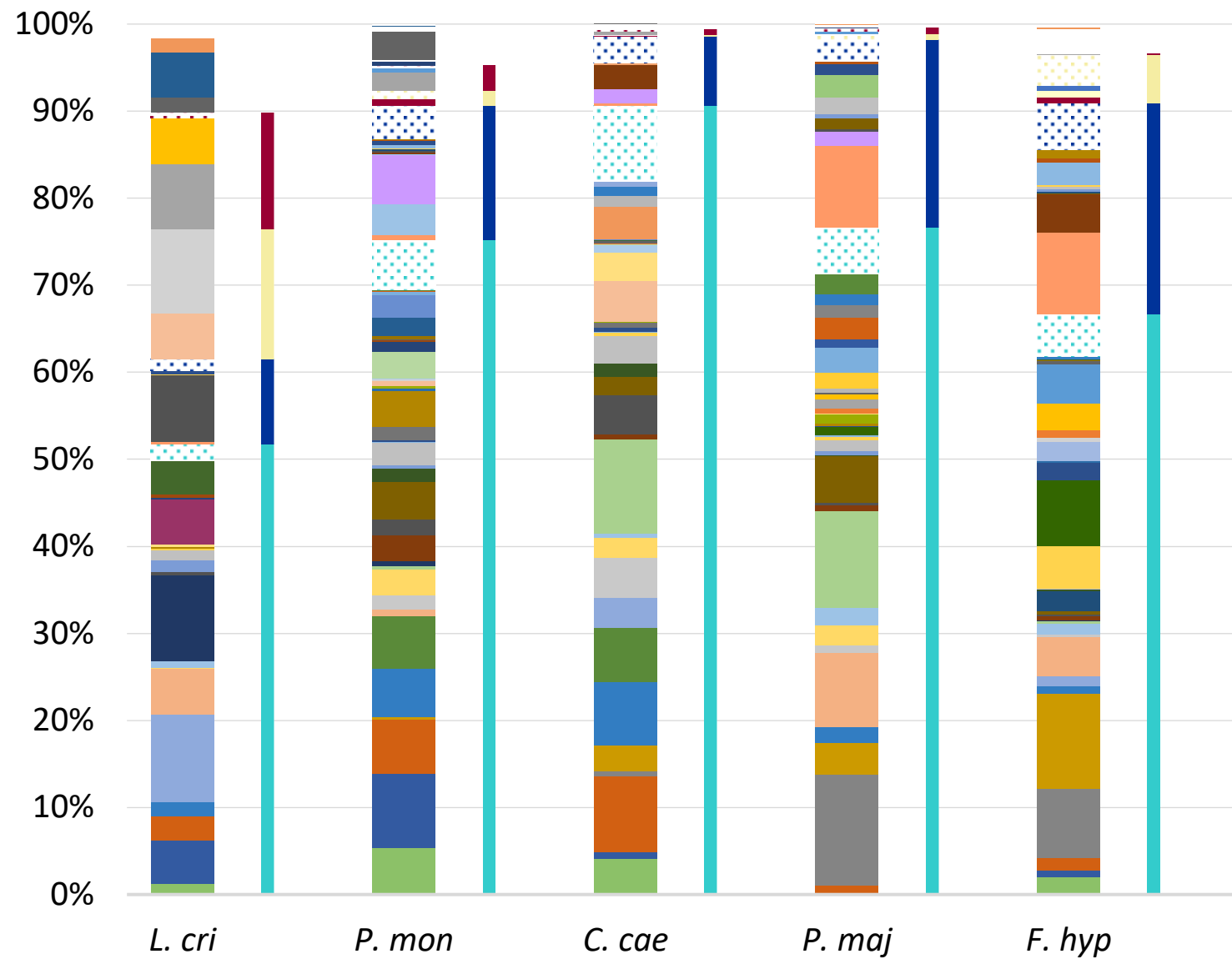

Figure S2. Mean yearly diets expressed as relative read abundances (RRA). The main bars display the 100 most abundant prey overall, grouped by order, in descending order of overall abundance. Patterned bar segments indicate sums of remaining species in prominent orders. Corresponding orders are shown as narrow bars to their right. Legend omitted due to the excessive length; order and colours match Fig. 1 and full details in Table S1.

MOLECULAR ECOLOGY

Table S1. Overview of the 100 most abundant prey species (or lowest-level identities available) in the birds' diets, in descending order (relative read abundances) of taxonomic orders, then descending order of abundance of species within each. Means 2018–2020. No value listed if fully absent. Coloured cells indicate lepidopterans available as larvae during the relevant time windows. Abbreviated orders indicate: Le = Lepidoptera, Di = Diptera, Co = Coleoptera, Ar = Araneae, Hy = Hymenoptera, He = Hemiptera, Si = Siphonaptera, Bl = Blattodea, Ps = Psocodea, Me = Megaloptera, Ne = Neuroptera, Sa = Sarcoptiformes, Tr = Trombidiformes, Tr = Trichoptera, Od = Odonata, Or = Orthoptera, Op = Opiliones.

Order	Family	Species	*L. cri*	*P. mon*	*C. cae*	*P. maj*	*F. hyp*
Le	Geometridae	*Epirrita autumnata*	3.61%	15.27%	10.05%	3.33%	0.94%
Le	Noctuidae	*Hyppa rectilinea*	13.66%	6.71%	2.00%	6.50%	0.70%
Le	Geometridae	*Operophtera brumata*	3.15%	5.05%	13.87%	6.01%	0.72%
Le	Geometridae	*Bupalus piniarius*	0.67%	1.43%	1.04%	7.75%	5.11%
Le	Tortricidae	*Epinotia crenana*	0.72%	0.38%	1.38%	2.83%	9.80%
Le	Geometridae	*Hydriomena furcata*	0.98%	2.98%	6.06%	4.26%	0.68%
Le	Geometridae	*Geometra papilionaria*	0.94%	8.02%	5.43%	0.50%	
Le	Noctuidae	*Panolis flammea*	5.70%	0.12%	5.56%		2.48%
Le	Noctuidae	*Orthosia gothica*	3.10%	0.29%	0.23%	3.40%	4.17%
Le	Noctuidae	*Brachylomia viminalis*	1.75%	0.88%	6.23%	1.46%	0.18%
Le	Ypsolophidae	*Ypsolopha parenthesella*	0.48%	2.21%	4.48%	2.22%	0.93%
Le	Geometridae	*Ectropis crepuscularia*	4.88%	0.03%	1.75%	1.45%	1.39%
Le	Noctuidae	*Enargia paleacea*		0.14%	3.66%	3.72%	0.16%
Le	Geometridae	*Hylaea fasciaria*	5.76%	1.52%		0.03%	0.01%
Le	Tortricidae	*Adoxophyes orana*	0.34%	3.10%	1.89%	0.70%	0.27%
Le	Noctuidae	*Orthosia incerta*	0.62%	0.73%	1.79%	0.39%	2.63%
Le	Geometridae	*Ematurga atomaria*	0.09%	2.69%	0.83%	2.15%	0.38%
Le	Geometridae	*Cabera pusaria*	0.14%	0.01%	2.14%	2.01%	1.68%
Le	Tortricidae	*Pandemis cerasana*	0.15%	2.80%	2.33%	0.07%	0.04%
Le	Tortricidae	*Zelotherses unitana*	1.04%	1.18%	0.81%	1.94%	
Le	Geometridae	*Xanthorhoe annotinata*	4.60%	0.01%			
Le	Lasiocampidae	*Poecilocampa populi*	0.45%	1.19%	1.80%	0.79%	0.06%
Le	Tortricidae	*Exapate congelatella*	0.12%	0.07%	0.19%	0.19%	3.65%
Le	Geometridae	*Odontopera bidentata*	0.93%	0.19%	3.05%		
Le	Noctuidae	*Conistra vaccinii*	0.90%	0.01%	0.03%	0.32%	2.63%
Le	Geometridae	*Macaria loricaria*	0.58%	0.48%	1.39%	0.54%	0.78%
Le	Erebidae	*Phragmatobia fuliginosa*	3.18%			0.02%	
Le	Tortricidae	*Epinotia solandriana*	0.09%	0.91%	1.97%	0.12%	
Le	Noctuidae	*Mniotype adusta*	0.17%	1.47%		0.96%	0.48%
Le	Coleophoridae	*Coleophora serratella*		2.49%	0.02%		0.08%
Le	Geometridae	*Crocallis elinguaria*	0.16%	0.37%	0.20%	1.73%	
Le	Geometridae	*Cleora cinctaria*	0.05%	0.03%	0.04%	0.27%	2.06%
Le	Geometridae	*Operophtera fagata*		0.22%	2.13%		
Le	Geometridae	*Eulithis populata*	0.05%	0.08%	0.01%	0.07%	1.88%

Le	Noctuidae	*Brachionycha nubeculosa*	0.31%	0.12%	1.46%	0.03%	0.02%
Le	Tortricidae	*Epinotia cinereana*	1.01%	0.76%	0.12%		
Le	Eriocraniidae	*Eriocrania semipurpurella*	0.07%	1.60%	0.04%		0.18%
Le	Lypusidae	*Agnoea josephinae*	1.73%				0.01%
Le	Erebidae	*Scoliopteryx libatrix*	0.41%	0.05%	0.39%	0.26%	0.58%
Le	Geometridae	*Alcis repandatus*	1.20%			0.37%	0.07%
Le	Geometridae	*Eupithecia lanceata*		0.14%	0.17%	1.31%	
Le	Notodontidae	*Notodonta dromedarius*	0.01%	0.02%	0.06%	1.48%	
Le	Geometridae	*Scopula floslactata*	1.52%				0.01%
Le	Notodontidae	*Pheosia gnoma*	0.03%	0.37%	0.01%	1.08%	
Le	Tortricidae	*Zeiraphera ratzeburgiana*	0.35%	1.08%			
Le	Lycaenidae	*Callophrys rubi*	0.66%	0.05%	0.11%	0.06%	0.52%
Le	Tortricidae	*Pandemis cinnamomeana*	0.42%	0.45%	0.40%	0.08%	0.03%
Le	Geometridae	*Arichanna melanaria*		0.80%		0.55%	
Le	Endromidae	*Endromis versicolora*	1.26%		0.02%		0.03%
Le	Noctuidae	*Coranarta cordigera*	0.04%	0.99%	0.02%	0.01%	0.21%
Le	Noctuidae	*Cosmia trapezina*			1.28%		
Le	Depressariidae	*Agonopterix conterminella*		0.08%	0.98%	0.15%	
Le	Nymphalidae	*Polygonia c-album*			1.15%		
Le	Notodontidae	*Ptilodon capucinus*	0.11%	0.02%	0.98%	0.01%	
Le	Geometridae	*Scopula ternata*	0.09%			0.02%	1.01%
Le	Nymphalidae	*Speyeria aglaja*	0.23%			0.88%	
Le	Lycaenidae	*Agriades optilete*		0.06%		0.99%	
Le	Nymphalidae	*Aglais urticae*		0.22%	0.79%		
Le	Tortricidae	*Epinotia nisella*		0.88%			
Le	Drepanidae	*Achlya flavicornis*		0.34%	0.41%	0.08%	
Le	Saturniidae	*Saturnia pavonia*	0.02%		0.79%		
Le	Gelechiidae	*Anacampsis blattariella*		0.04%	0.22%	0.30%	0.25%
Le	Geometridae	*Cabera exanthemata*	0.04%	0.02%			0.69%
Le		Sum other Lepidoptera	3.53%	4.72%	5.21%	6.85%	5.43%
Di	Rhagionidae	*Rhagio scolopaceus*	1.28%	0.73%	0.17%	6.46%	13.28%
Di	Tipulidae	*Tipula sintenisi*	0.34%	4.79%	0.01%	2.55%	0.04%
Di	Tachinidae	*Smidtia amoena*	0.17%	4.88%	0.57%	1.11%	0.32%
Di	Simuliidae	*Simulium sp.*	1.90%	1.32%	1.38%		
Di	Bibionidae	*Bibio nigriventris*	0.44%	0.21%	1.26%	0.35%	2.16%
Di	Tachinidae	*Ramonda ringdahli*	3.93%	0.06%		0.11%	0.01%
Di	Tipulidae	*Tipula lunata*	0.22%	0.42%	0.01%	2.23%	0.80%
Di	Cecidomyiidae	*(Family Cecidomyiidae)*	0.59%	0.07%	0.40%	1.01%	0.15%
Di	Phoridae	*Megaselia lucifrons*	1.40%				
Di	Dolichopodidae	*Dolichopus discifer*			0.15%	1.21%	
Di	Syrphidae	*Syrphus vitripennis*	0.09%	0.06%	0.06%		0.97%

MOLECULAR ECOLOGY

Di	Tachinidae	*Oswaldia muscaria*	0.07%		0.63%	0.45%	
Di	Culicidae	*Ochlerotatus sp.*	0.05%	0.86%		0.07%	0.14%
Di	Limoniidae	*(Family Limoniidae)*	0.16%	0.01%		0.95%	
Di	Tabanidae	*Hybomitra sp.*	0.11%		0.86%	0.12%	
Di	Pediciidae	*Pedicia rivosa*	0.12%	0.29%	0.01%	0.64%	
Di	Tabanidae	*Hybomitra bimaculata*	0.16%	0.03%		0.27%	0.46%
Di	Tipulidae	*Tipula pierrei*			0.82%		
Di		*(Order Diptera)*	0.04%	0.05%	0.05%	0.05%	0.61%
Di	Tachinidae	*Hyalurgus crucigerus*	0.06%			0.72%	
Di		Sum other Diptera	1.22%	3.01%	2.14%	3.49%	4.92%
Co	Carabidae	*Pterostichus oblongopunctatus*	0.34%	0.26%	0.03%		5.64%
Co	Cerambycidae	*Rhagium mordax*	3.11%	0.03%	0.01%	0.72%	
Co	Cerambycidae	*Rhagium inquisitor*	3.34%				
Co	Carabidae	*Pterostichus sp.*	0.22%		0.06%		1.41%
Co	Staphylinidae	*Atheta myrmecobia*	0.20%	0.25%	0.04%	0.40%	0.23%
Co	Staphylinidae	*Anthophagus omalinus*	0.25%	0.17%	0.20%	0.21%	0.28%
Co	Tenebrionidae	*Lagria hirta*		0.08%		0.87%	
Co		Sum other Coleoptera	0.37%	0.17%	0.59%	1.81%	
Ar	Philodromidae	*Philodromus cespitum*	2.64%	0.84%	0.13%		0.05%
Ar	Salticidae	*Evarcha sp.*	1.75%				
Ar	Philodromidae	*Tibellus sp.*	0.16%	0.17%	0.37%	0.05%	
Ar		Sum other Araneae	0.58%	0.40%	0.16%	0.33%	0.14%
Hy	Vespidae	*Dolichovespula norwegica*	0.03%	1.40%		0.67%	
Hy		Sum other Hymenoptera	0.33%	0.62%	0.22%	0.29%	1.40%
He	Aphididae	*Cinara pruinosa*	0.58%	1.51%	0.22%	0.01%	
He		Sum other Hemiptera	0.11%	0.49%	0.12%	0.05%	0.32%
Si	Ceratophyllidae	*Ceratophyllus sp.*	1.87%	0.31%		0.32%	1.02%
Si		Sum other Siphonaptera	0.01%				
Bl	Ectobiidae	*Ectobius sylvestris*	0.25%	0.26%		0.05%	2.38%
Ps	Caeciliusidae	*Valenzuela flavida*	0.81%		0.05%	0.04%	0.05%
Ps	Caeciliusidae	*Valenzuela despaxi*	0.75%				
Me	Sialidae	*Sialis fuliginosa*	0.19%		0.02%		0.67%
Ne		Sum Neuroptera	0.10%	0.12%		0.09%	0.08%
Sa		Sum Sarcoptiformes	0.23%	0.04%		0.01%	0.02%
Tr		Sum Trombidiformes	0.10%	0.01%	0.04%		
Tr		Sum Trichoptera	0.03%			0.08%	
Od		Sum Odonata				0.01%	
Or		Sum Orthoptera			0.01%		
Op		Sum Opiliones				0.01%	

MOLECULAR ECOLOGY

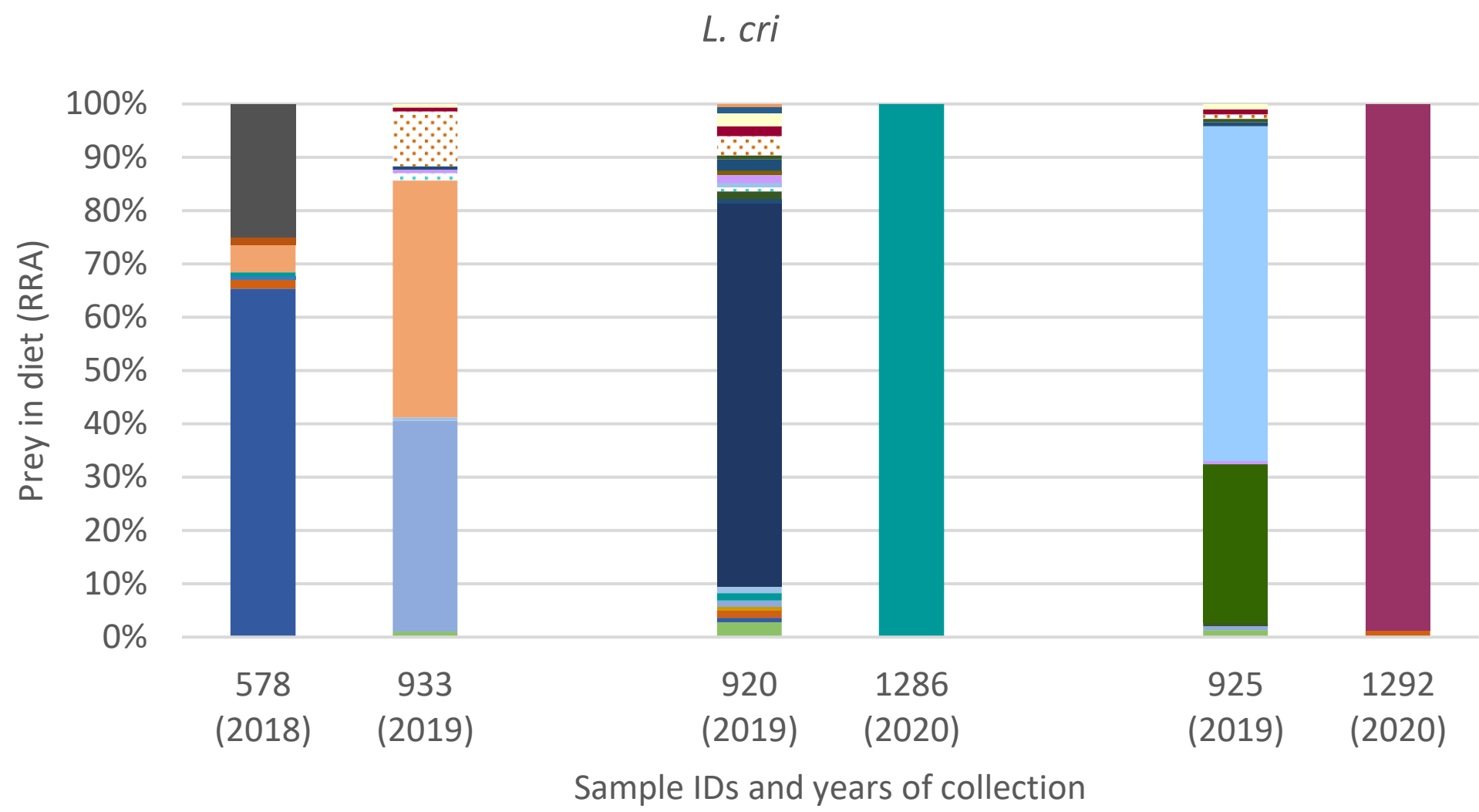

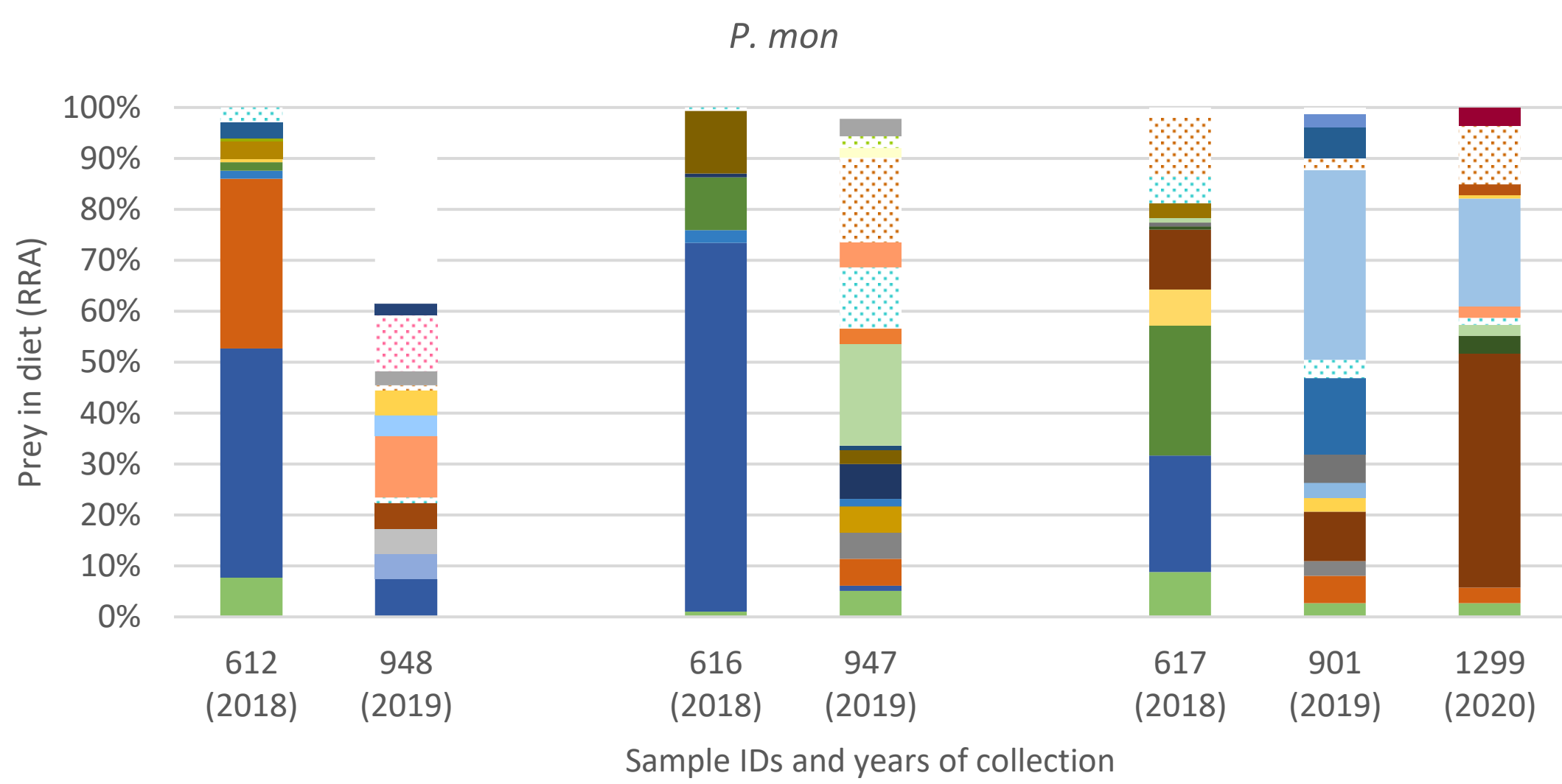

MOLECULAR ECOLOGY

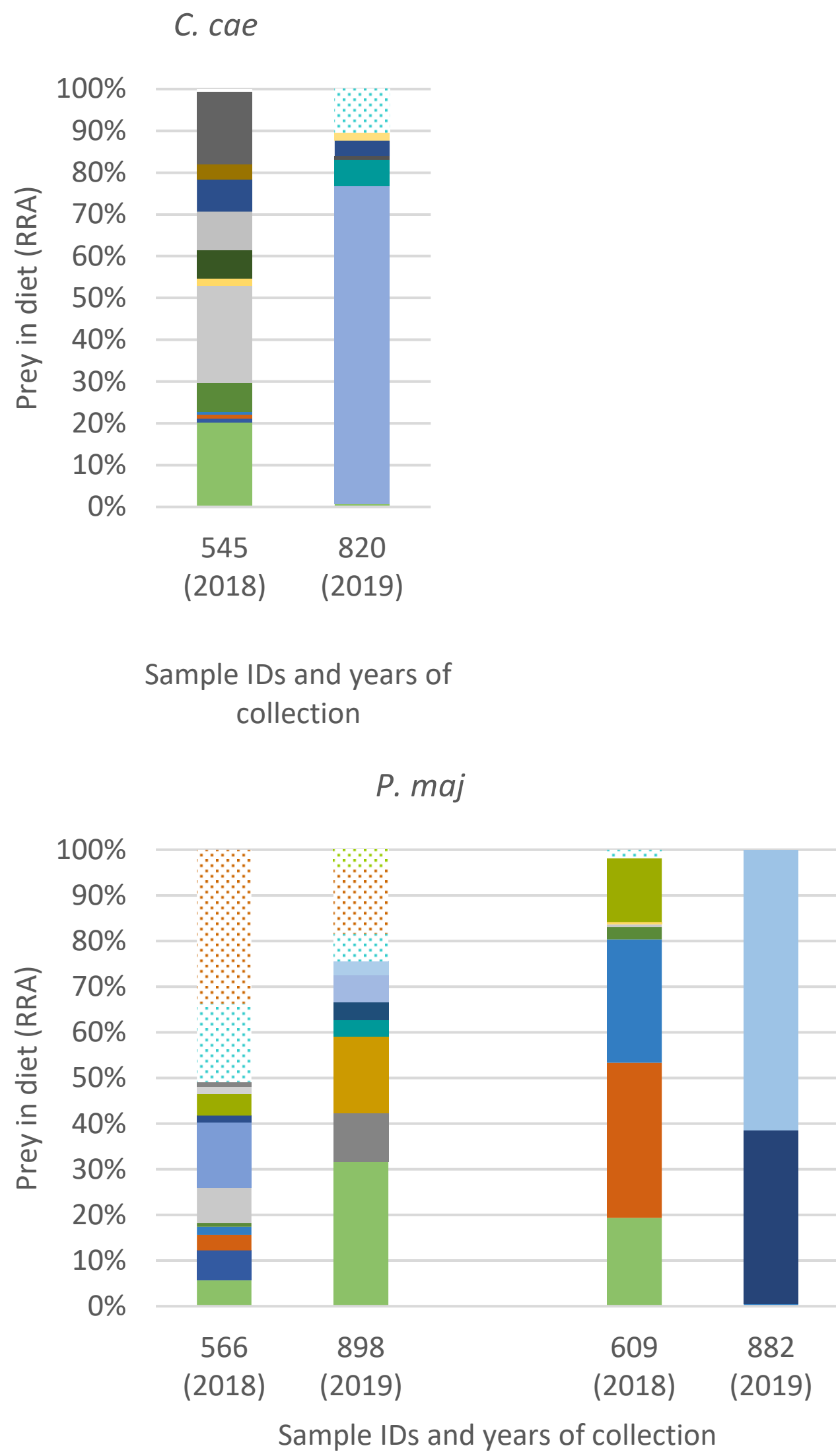

Figure S3. Diets of repeated parents, separated by species. Mean diets expressed as relative read abundances (RRA). Displayed are the 100 most abundant prey across the data as per Figure 1 and Figure S2, SI2 (in matching colours), grouped by order, in descending order of overall abundance. Patterned bar segments indicate sums of remaining species in prominent orders. Pairs or trios of closely neighbouring bars represent the same pair of parents, nesting at the same location (i.e., the same nest box/a generally newly excavated cavity within 100 m of a previous year's nest).

Additional results: Most shared prey in samples from repeated parents were commonly consumed by the bird species in general (*Epirrita autumnata*, *Hyppa rectilinea, Operophtera brumata*), with abundances varying substantially between repeated samples. Otherwise: *Xanthorhoe annotinata* was found at 5% and 44% in two crested tit samples; it occurred >30% in 2 out of 10 other crested tit samples from the latter's year. *Adoxophyes orana* was prominent in willow tit samples from three years; it was also present in 12, 4, and 5 conspecific samples from 2018, 2019, and 2020 respectively. Two of the same three samples contained *Tipula sintenisi* (with an absence for 2018), which was found in 4, 8, and 2 other willow tit samples from 2018, 2019, and 2020 respectively.

MOLECULAR ECOLOGY

2018

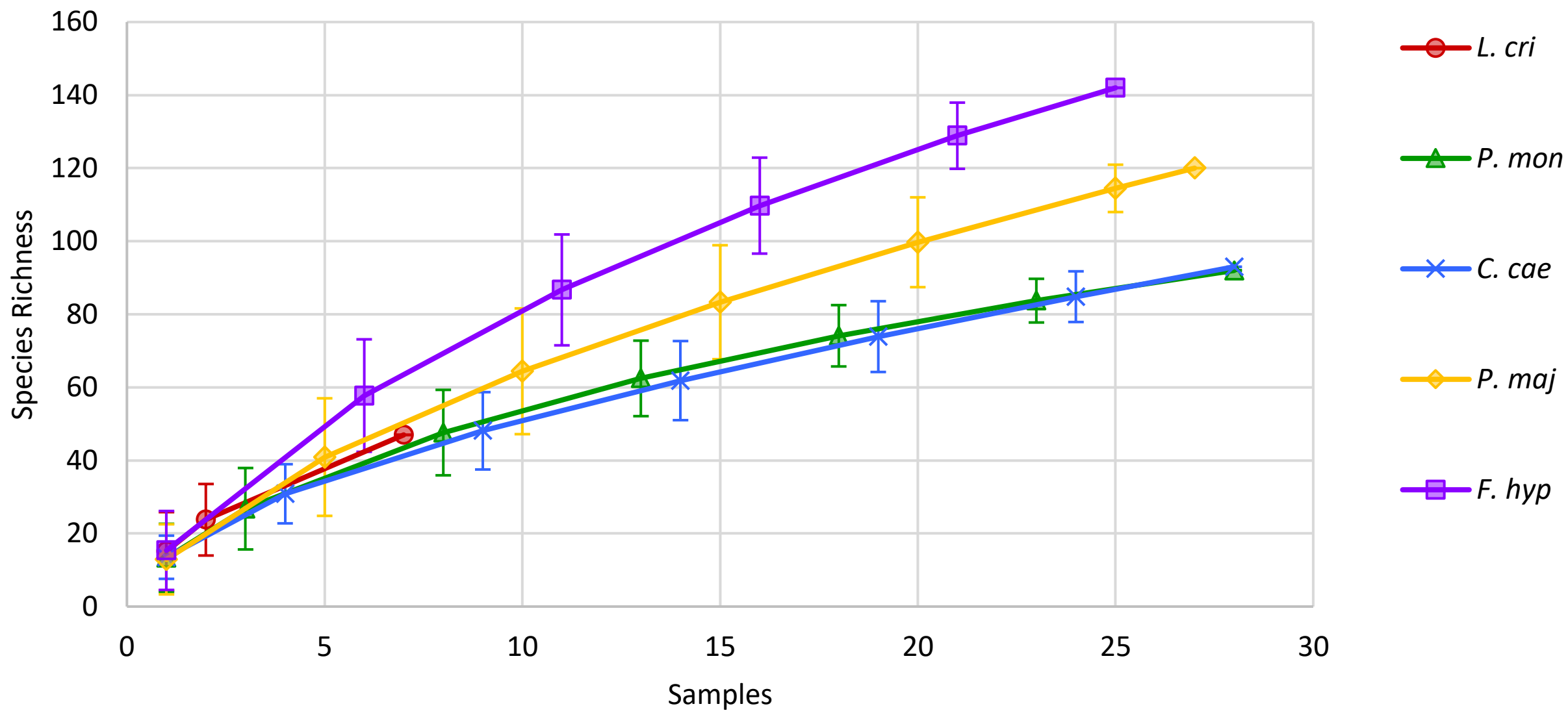

2019

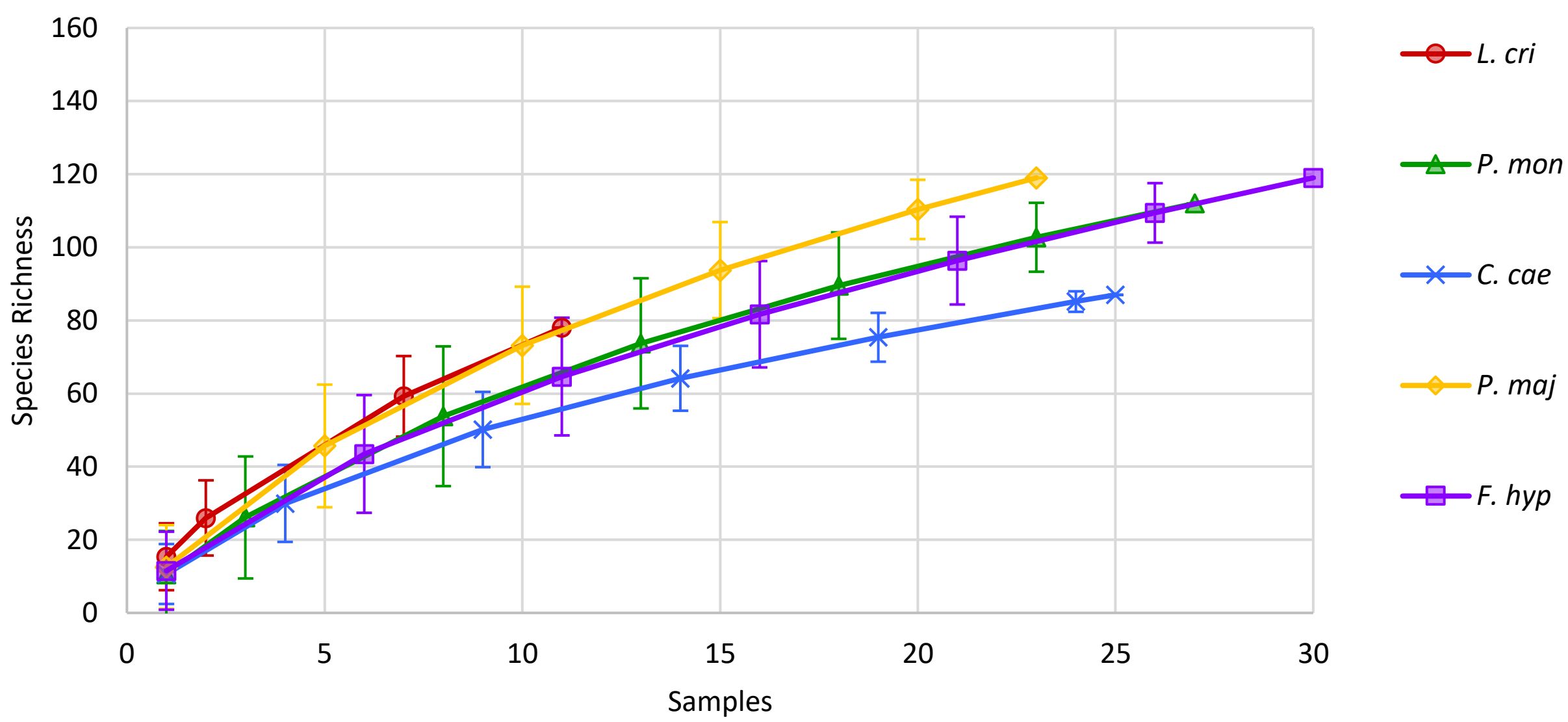

MOLECULAR ECOLOGY

2020

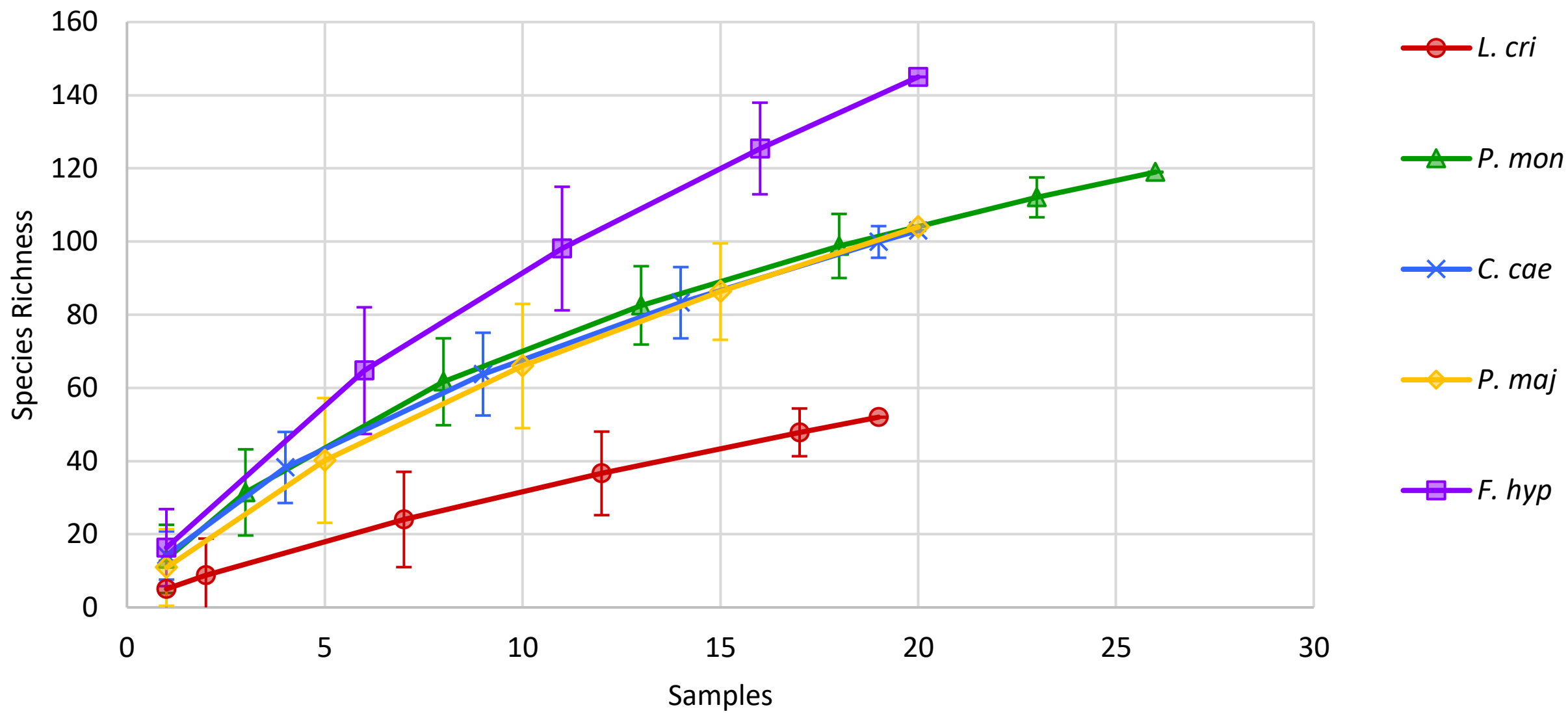

Figure S4. Species accumulation curves for the individual years. Error bars display 2× SD. Only the first, last, and otherwise every fifth sample were plotted (at different intervals for the different bird species) for improved discernability.

MOLECULAR ECOLOGY

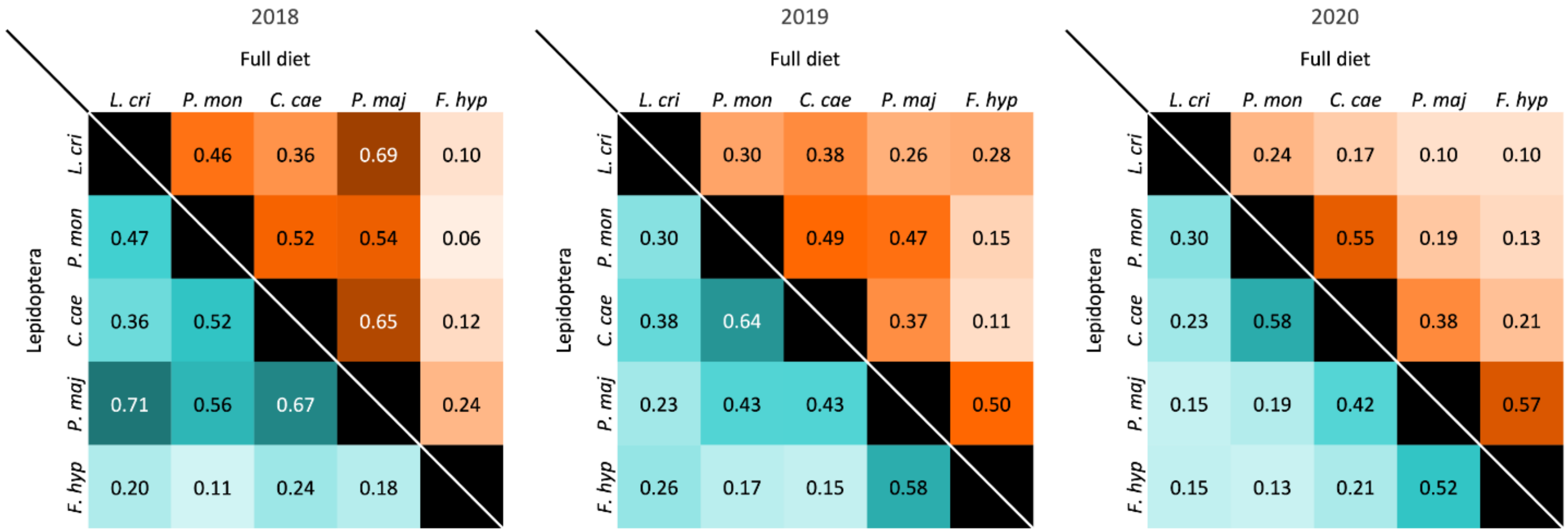

Figure S5. Pianka’s niche overlap scores for the sampled years. The top right shows the overall overlap for the full diets, the lower left shows scores for the Lepidoptera in the diets only; darker colours indicate higher overlap in either. White numbers indicate values >0.6.

MOLECULAR ECOLOGY

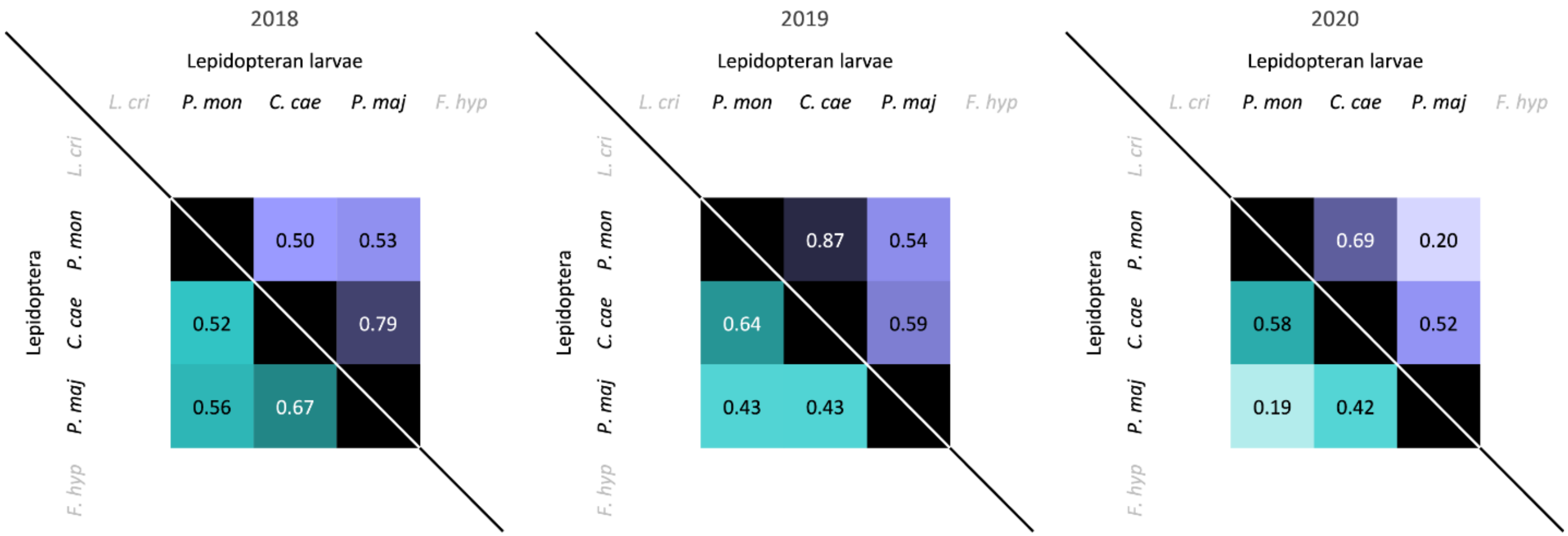

Figure S6. Pianka's niche overlap scores for the sampled years. The lower left shows the overlap for the Lepidoptera in the diets, the top right shows the scores for the lepidopteran larvae only; darker colours indicate higher overlap in either. White numbers indicate values >0.6.

MOLECULAR ECOLOGY

Table S2. Pearson correlations between the PCOs and the explanatory variables. For brevity, only PCOs with explanatory value >2.5% are listed. The percentages given in square brackets below each PCO correspond to the variation explained by each.

Variable	PCO1 [11.2%]	PCO2 [5.8%]	PCO3 [5.6%]	PCO4 [4.8%]	PCO5 [4.1%]	PCO6 [3.7%]	PCO7 [3.1%]	PCO8 [2.8%]	PCO9 [2.7%]
Free-living	0.481	0.014	−0.185	−0.027	−0.048	0.069	0.104	0.226	−0.038
Mining	0.023	0.033	0.014	−0.054	−0.075	−0.082	0.040	0.009	−0.147
Rolling	0.308	0.532	−0.295	0.082	−0.038	−0.204	0.403	0.030	0.137
Webbing	0.118	0.144	0.092	−0.035	−0.242	−0.257	−0.138	0.160	0.409
Casing	−0.018	−0.007	0.027	−0.043	0.007	0.019	−0.012	−0.036	−0.010
Urticating setae	0.121	0.139	0.072	−0.004	−0.106	−0.121	−0.101	0.039	0.104
Canopy	0.558	0.072	−0.192	−0.057	−0.124	−0.064	0.132	0.259	0.052
Birch	0.374	−0.266	−0.064	−0.128	−0.265	−0.292	−0.050	0.144	0.192
Deciduous	0.537	0.096	−0.286	0.093	−0.190	−0.199	0.292	0.058	0.128
Coniferous	−0.216	−0.090	0.030	−0.187	0.222	0.154	0.056	0.363	−0.003
Egg	−0.140	0.007	0.099	−0.195	0.318	−0.087	−0.029	0.150	−0.104
Larva	0.414	0.073	−0.279	0.006	−0.101	−0.125	0.327	0.205	0.111
Pupa	0.099	−0.025	0.633	0.482	0.074	−0.059	0.036	−0.020	−0.083
Imago	−0.097	−0.050	0.151	−0.298	0.274	0.015	−0.058	0.102	−0.099
Year	−0.248	0.027	0.028	−0.317	0.111	0.049	0.056	−0.169	−0.038
May date	−0.487	0.044	−0.203	0.020	−0.081	−0.068	0.014	0.069	0.088
Sequencing run	−0.239	0.025	0.096	−0.301	0.082	0.080	0.070	−0.152	−0.062
Latitude	−0.145	−0.148	0.149	0.079	0.060	−0.001	0.132	−0.090	−0.017

MOLECULAR ECOLOGY

Table S3. Results of marginal tests for DistLM solutions for all prey species and sample variables.

Variable	SS (trace)	Pseudo-F	P-value	Proportion of variation explained
Free-living	50261	11.83	≤0.001	0.034
Mining	8140.2	1.8608	0.002	0.006
Rolling	60973	14.461	≤0.001	0.041
Webbing	33842	7.8746	≤0.001	0.023
Casing	5497.1	1.2543	0.038	0.004
Urticating setae	17370	3.9959	≤0.001	0.012
Canopy	64138	15.246	≤0.001	0.044
Birch	48089	11.302	≤0.001	0.033
Deciduous	68842	16.419	≤0.001	0.047
Coniferous	25083	5.801	≤0.001	0.017
Egg	19741	4.5487	≤0.001	0.013
Larva	47857	11.246	≤0.001	0.033
Pupa	54343	12.828	≤0.001	0.037
Imago	22023	5.0826	≤0.001	0.015
Year	25027	5.7878	≤0.001	0.017
May date	49001	11.524	≤0.001	0.033
Sequencing run	23294	5.3806	≤0.001	0.016
Latitude	15187	3.4885	≤0.001	0.010

MOLECULAR ECOLOGY

Table S4. Best DistLM solutions for different numbers and combinations of variables, based on both lowest AICc and BIC scores. Listed are both the best models for each number of variables and the overall best solutions with the lowest scores as well as any scores within 1. ΔAICc/BIC refers to the difference in information criterion score compared to the lowest value obtained for that metric. Best solutions for either metric in bold text; results with scores within 1 as underlined text (AICc) or italics (BIC). Variables: 1 = Free-living, 2 = Mining, 3 = Rolling, 4 = Webbing, 5 = Casing, 6 = Urticating setae, 7 = Canopy, 8 = Birch, 9 = Deciduous, 10 = Coniferous, 11 = Egg, 12 = Larva, 13 = Pupa, 14 = Imago, 15 = Year, 16 = May date, 17 = Sequencing run, 18= Latitude.

Selected variables	AICc	ΔAICc	BIC	ΔBIC	R^2	Residual Sum of Squares	Number of variables
9	2804.7	76.3	2812.2	32.8	0.047	1.400E6	1
9, 13	2793.2	64.8	2804.6	25.2	0.084	1.345E6	2
9, 13, 16	2783.4	55.0	2798.5	19.1	0.116	1.299E6	3
3, 9, 13, 16	2773.4	45.0	2792.3	12.9	0.147	1.253E6	4
3, 7, 9, 10, 13	2764.9	36.5	2787.5	8.1	0.174	1.214E6	5
3, 7, 9, 10, 13, 16	2757.6	29.2	2784.0	4.6	0.196	1.181E6	6
3, 4, 7, 9, 10, 13, 16	2750.9	22.5	2781.0	1.6	0.217	1.150E6	7
3, 4, 7–10, 13, 16	2745.9	17.5	*2779.7*	0.3	0.234	1.126E6	8
3, 4, 7–11, 13, 16	2741.9	13.5	***2779.4***	***0.0***	0.247	1.106E6	9
3, 4, 7–10, 13, 14, 16	2742.0	13.6	*2779.5*	0.1	*0.247*	*1.106E6*	*9*
1, 3, 4, 7–11, 13, 16	2738.8	10.4	*2780.0*	0.6	0.259	1.089E6	10
1, 3, 4, 7–10, 13, 14, 16	2738.9	10.5	*2780.1*	0.7	*0.259*	*1.089E6*	*10*
1, 3, 4, 7–11, 13, 15, 16	2736.2	7.8	2781.0	1.6	0.270	1.073E6	11
1, 3, 4, 7–11, 13-16	2733.7	5.3	2782.2	2.8	0.280	1.058E6	12
1, 3, 4, 6–11, 13-16	2732.0	3.6	2784.1	4.7	0.288	1.046E6	13
1, 3, 4, 6–16	2730.4	2.0	2786.1	6.7	0.296	1.035E6	14
1–4, 6–16	2729.2	0.8	2788.6	9.2	0.303	1.024E6	15
1–4, 6–14, 16, 17	2729.3	0.9	2788.7	9.3	0.303	1.025E6	15
**1–16**	**2728.4**	**0.0**	2791.4	12.0	0.309	1.015E6	16
1–14, 16, 17	2728.5	0.1	2791.4	12.0	0.309	1.015E6	16
1–4, 6–16, 18	2729.2	0.8	2792.2	12.8	0.307	1.018E6	16
1–4, 6–14, 16–18	2729.3	0.9	2792.3	12.9	0.307	1.018E6	16
1–16, 18	2728.4	0.0	2795.0	15.6	0.314	1.008E6	17
1–14, 16–18	2728.5	0.1	2795.1	15.7	0.314	1.009E6	17
1–17	2729.1	0.7	2795.7	16.3	0.312	1.011E6	17
1–18 (= all)	2729.2	0.8	2799.3	19.9	0.317	1.004E6	18

MOLECULAR ECOLOGY

Table S5. Best DistLM solutions (lowest BIC and scores within 1) for pairwise comparisons of the bird species. Variables: 1 = Free-living, 2 = Mining, 3 = Rolling, 4 = Webbing, 5 = Casing, 6 = Urticating setae, 7 = Canopy, 8 = Birch, 9 = Deciduous, 10 = Coniferous, 11 = Egg, 12 = Larva, 13 = Pupa, 14 = Imago, 15 = Year, 16 = May date, 17 = Sequencing run, 18= Latitude. Also listed are the best PCO axes and the percentages of variation explained by each.

Compared species		Selected variables	BIC	R^2	Residual Sum of Squares	Number of variables	PCO1	PCO2
		9, 13	983.2	0.129	4.344E5	2		
L. cri	*P. mon*	8, 9, 13	983.9	0.159	4.194E5	3	11.8%	8.4%
		3, 9, 13	984.2	0.157	4.206E5	3		
		3, 9, 13	915.4	0.157	3.818E5	3		
		3, 4, 9, 13	915.4	0.192	3.654E5	4		
		3, 9	915.6	0.118	3.988E5	2		
		3, 13	915.7	0.117	3.991E5	2		
L. cri	*C. cae*	1, 3, 4, 10, 13	915.8	0.223	3.513E5	5	14.3%	8.2%
		3	915.8	0.078	4.168E5	1		
		3, 4, 13	915.9	0.153	3.830E5	3		
		3, 8, 13	915.9	0.153	3.832E5	3		
		3, 4, 9	916.0	0.152	3.832E5	3		
		1, 3, 10, 13	916.1	0.187	3.675E5	4		
L. cri	*P. maj*	3, 13	899.9	0.117	4.217E5	2	9.1%	8.2%
		3, 10, 13	900.9	0.147	4.074E5	3		
		3, 16	937.2	0.116	4.252E5	2		
L. cri	*F. hyp*	3	937.6	0.075	4.449E5	1	11.7%	6.2%
		3, 10, 16	937.8	0.148	4.097E5	3		
		1, 3, 4, 8, 12, 13, 16	1253.5	0.324	4.065E5	7		
		1, 3–5, 8, 12, 13, 16	1253.5	0.324	4.065E5	8		
		1, 3, 4, 8–10, 13, 16	1253.6	0.345	3.936E5	8		
		1, 3–5, 8–10, 13, 16	1253.6	0.345	3.936E5	9		
		3, 4, 8–10, 13, 16	1253.8	0.323	4.070E5	7		
		3–5, 8–10, 13, 16	1253.8	0.323	4.070E5	8		
		1, 3, 4, 8, 9, 13, 16	1253.8	0.323	4.071E5	7		
		1, 3–5, 8, 9, 13, 16	1253.8	0.323	4.071E5	8		
		3, 4, 7, 8, 13, 16	1253.9	0.300	4.210E5	6		
		3–5, 7, 8, 13, 16	1253.9	0.300	4.210E5	7		
		1, 3, 4, 8, 10, 12, 13, 16	1253.9	0.344	3.943E5	8		
		1, 3–5, 8, 10, 12, 13, 16	1253.9	0.344	3.943E5	9		
P. mon	*C. cae*	1, 3, 4, 8, 13, 16	1253.9	0.300	4.210E5	6	13.0%	9.3%
		1, 3–5, 8, 13, 16	1253.9	0.300	4.210E5	7		
		3, 4, 8, 12, 13, 16	1253.9	0.300	4.211E5	6		
		3–5, 8, 12, 13, 16	1253.9	0.300	4.211E5	7		
		1, 3, 4, 8, 9, 12, 13, 16	1254.0	0.344	3.944E5	8		
		1, 3–5, 8, 9, 12, 13, 16	1254.0	0.344	3.944E5	9		
		1, 3, 4, 7, 8, 13, 16	1254.2	0.321	4.082E5	7		
		1, 3–5, 7, 8, 13, 16	1254.2	0.321	4.082E5	8		
		3, 4, 7, 8, 10, 13, 16	1254.2	0.321	4.083E5	7		
		3–5, 7, 8, 10 ,13, 16	1254.2	0.321	4.083E5	8		
		1–4, 8, 12, 13, 16	1254.3	0.342	3.953E5	8		
		1–5, 8, 12, 13, 16	1254.3	0.342	3.953E5	9		

MOLECULAR ECOLOGY

		3, 4, 8, 10, 12, 13, 16	1254.4	0.320	4.086E5	7		
		3–5, 8, 10, 12, 13, 16	1254.4	0.320	4.086E5	8		
		1–4, 8–10, 13, 16	1254.4	0.363	3.827E5	9		
		1–5, 8–10, 13, 16	1254.4	0.363	3.827E5	10		
		3, 4, 8, 9, 12, 13, 16	1254.4	0.320	4.086E5	7		
		3–5, 8, 9, 12, 13, 16	1254.4	0.320	4.086E5	8		
		3, 4, 8, 9, 13, 16	1254.4	0.297	4.223E5	6		
		3–5, 8, 9, 13, 16	1254.4	0.297	4.223E5	7		
		1, 3, 4, 8, 10, 13, 16	1254.4	0.320	4.088E5	7		
		1, 3–5, 8, 10, 13, 16	1254.4	0.320	4.088E5	8		
		1, 3, 4, 8–10, 13, 14, 16	1254.4	0.363	3.829E5	9		
		1, 3–5, 8–10, 13, 14, 16	1254.4	0.363	3.829E5	10		
P. mon	*P. maj*	3, 8, 9, 11, 13, 16	1249.1	0.269	4.683E5	6	11.3%	7.9%
		3, 5, 8, 9, 11, 13, 16	1249.1	0.269	4.683E5	7		
		3, 7, 8, 11, 13, 16	1249.2	0.269	4.686E5	6		
		3, 5, 7, 8, 11, 13, 16	1249.2	0.269	4.686E5	7		
		3, 4, 8, 9, 11, 13, 16	1249.5	0.291	4.542E5	7		
		3–5, 8, 9, 11, 13, 16	1249.5	0.291	4.542E5	8		
		3, 4, 7, 8, 11, 13, 16	1249.6	0.291	4.546E5	7		
		3–5, 7, 8, 11, 13, 16	1249.6	0.291	4.546E5	8		
		3, 8, 11–13, 16	1249.7	0.267	4.701E5	6		
		3, 5, 8, 11–13, 16	1249.7	0.267	4.701E5	7		
		3, 9, 11, 13, 16	1249.9	0.241	4.867E5	5		
		3, 5, 9, 11, 13, 16	1249.9	0.241	4.867E5	6		
		1, 3, 8, 11–13, 16	1249.9	0.289	4.555E5	7		
		1, 3, 5, 8, 11–13, 16	1249.9	0.289	4.555E5	8		
		3, 7, 11, 13, 16	1250.0	0.240	4.870E5	5		
		3, 5, 7, 11, 13, 16	1250.0	0.240	4.870E5	6		
		3, 8, 9, 13, 14, 16	1250.0	0.265	4.711E5	6		
		3, 5, 8, 9, 13, 14, 16	1250.0	0.265	4.711E5	7		
		3, 4, 8, 11–13, 16	1250.0	0.289	4.558E5	7		
		3–5, 8, 11–13, 16	1250.0	0.289	4.558E5	8		
P. mon	*F. hyp*	7, 9, 10, 13, 16	1296.3	0.223	5.219E5	5	14.7%	6.2%
		5, 7, 9, 10, 13, 16	1296.3	0.223	5.219E5	6		
		7, 9, 10, 16	1296.4	0.197	5.395E5	4		
		5, 7, 9, 10, 16	1296.4	0.197	5.395E5	5		
		7, 10, 12, 13, 16	1297.2	0.219	5.249E5	5		
		5, 7, 10, 12, 13, 16	1297.2	0.219	5.249E5	6		
		7–10, 13, 16	1297.2	0.244	5.082E5	6		
		5, 7–10, 13, 16	1297.2	0.244	5.082E5	7		
C. cae	*P. maj*	1, 3, 4, 7, 9–11, 13, 16	1173.1	0.377	3.692E5	9	12.6%	8.0%
		1, 3–5, 7, 9–11, 13, 16	1173.1	0.377	3.692E5	10		
		1, 3, 4, 10–13, 16	1173.4	0.354	3.830E5	8		
		1, 3–5, 10–13, 16	1173.4	0.354	3.830E5	9		
		3, 4, 7–11, 13, 16	1173.4	0.376	3.700E5	9		
		3–5, 7–11, 13, 16	1173.4	0.376	3.700E5	10		
		1, 3, 4, 7–11, 13, 16	1173.5	0.397	3.578E5	10		
		1, 3–5, 7–11, 13, 16	1173.5	0.397	3.578E5	11		
		1, 3, 4, 7, 9–11, 13, 14, 16	1173.5	0.397	3.578E5	10		

MOLECULAR ECOLOGY

		1, 3–5, 7, 9–11, 13, 14, 16	1173.5	0.397	3.578E5	11		
		1, 3, 4, 9–11, 13, 16	1173.6	0.353	3.837E5	8		
		1, 3–5, 9–11, 13, 16	1173.6	0.353	3.837E5	9		
		1, 3, 4, 8, 10–13, 16	1173.7	0.374	3.709E5	9		
		1, 3–5, 8, 10–13, 16	1173.7	0.374	3.709E5	10		
		3, 4, 7, 9–11, 13, 16	1173.7	0.352	3.841E5	8		
		3–5, 7, 9–11, 13, 16	1173.7	0.352	3.841E5	9		
		1, 3, 4, 7, 9, 10, 13, 14, 16	1173.8	0.374	3.711E5	9		
		1, 3–5, 7, 9, 10, 13, 14, 16	1173.8	0.374	3.711E5	10		
		1, 3, 4, 7–11, 13, 14, 16	1173.9	0.416	3.464E5	11		
		1, 3–5, 7–11, 13, 14, 16	1173.9	0.416	3.464E5	12		
		3, 4, 7–11, 13, 14, 16	1173.9	0.395	3.587E5	10		
		3–5, 7–11, 13, 14, 16	1173.9	0.395	3.587E5	11		
		1, 3, 4, 10–14, 16	1174.0	0.373	3.715E5	9		
		1, 3–5, 10–14, 16	1174.0	0.373	3.715E5	10		
C. cae	*F. hyp*	3, 4, 7, 9, 10, 16	1219.8	0.291	4.437E5	6	16.2%	6.9%
		3–5, 7, 9, 10, 16	1219.8	0.291	4.437E5	7		
		3, 4, 7, 10, 12, 16	1220.5	0.288	4.457E5	6		
		3–5, 7, 10, 12, 16	1220.5	0.288	4.457E5	7		
P. maj	*F. hyp*	1, 3, 10, 12–14	1207.1	0.239	4.704E5	6	11.1%	8.4%
		1, 3, 5, 10, 12–14	1207.1	0.239	4.704E5	7		
		1, 3, 10–13	1207.7	0.236	4.722E5	6		
		1, 3, 5, 10–13	1207.7	0.236	4.722E5	7		
		1, 3, 12–14	1207.7	0.210	4.888E5	5		
		1, 3, 5, 12–14	1207.7	0.210	4.888E5	6		
		1, 3, 9, 12–14	1207.8	0.235	4.728E5	6		
		1, 3, 5, 9, 12–14	1207.8	0.235	4.728E5	7		
		1, 3, 11–13	1207.9	0.209	4.894E5	5		
		1, 3, 5, 11–13	1207.9	0.209	4.894E5	6		

MOLECULAR ECOLOGY

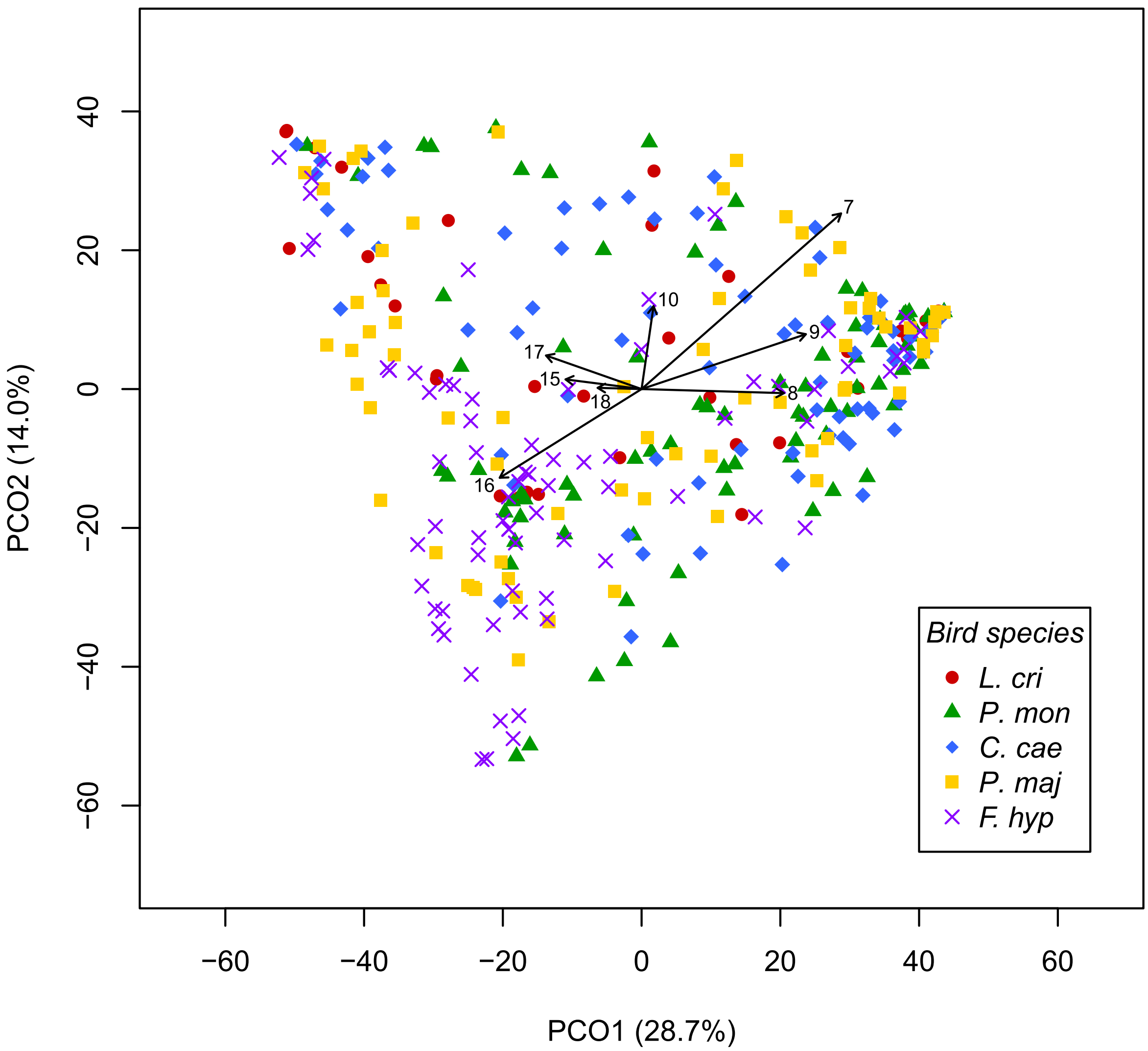

Figure S7. PCO ordinations of the bird species' diets analysed by prey family. Percentages next to the PCO axis titles refer to the percentage of variation explained by each. Vectors display Pearson correlations of prey traits and other variables, with relative lengths corresponding to their correlations to the axes. Only variables included in the best fitting DistLM models (lowest AICc or BIC, and scores within 1 of either) are shown (Table S6). Variables: 7 = Canopy, 8 = Birch, 9 = Deciduous, 10 = Coniferous, 15 = Year, 16 = May date, 17 = Sequencing run, 18= Latitude.

MOLECULAR ECOLOGY

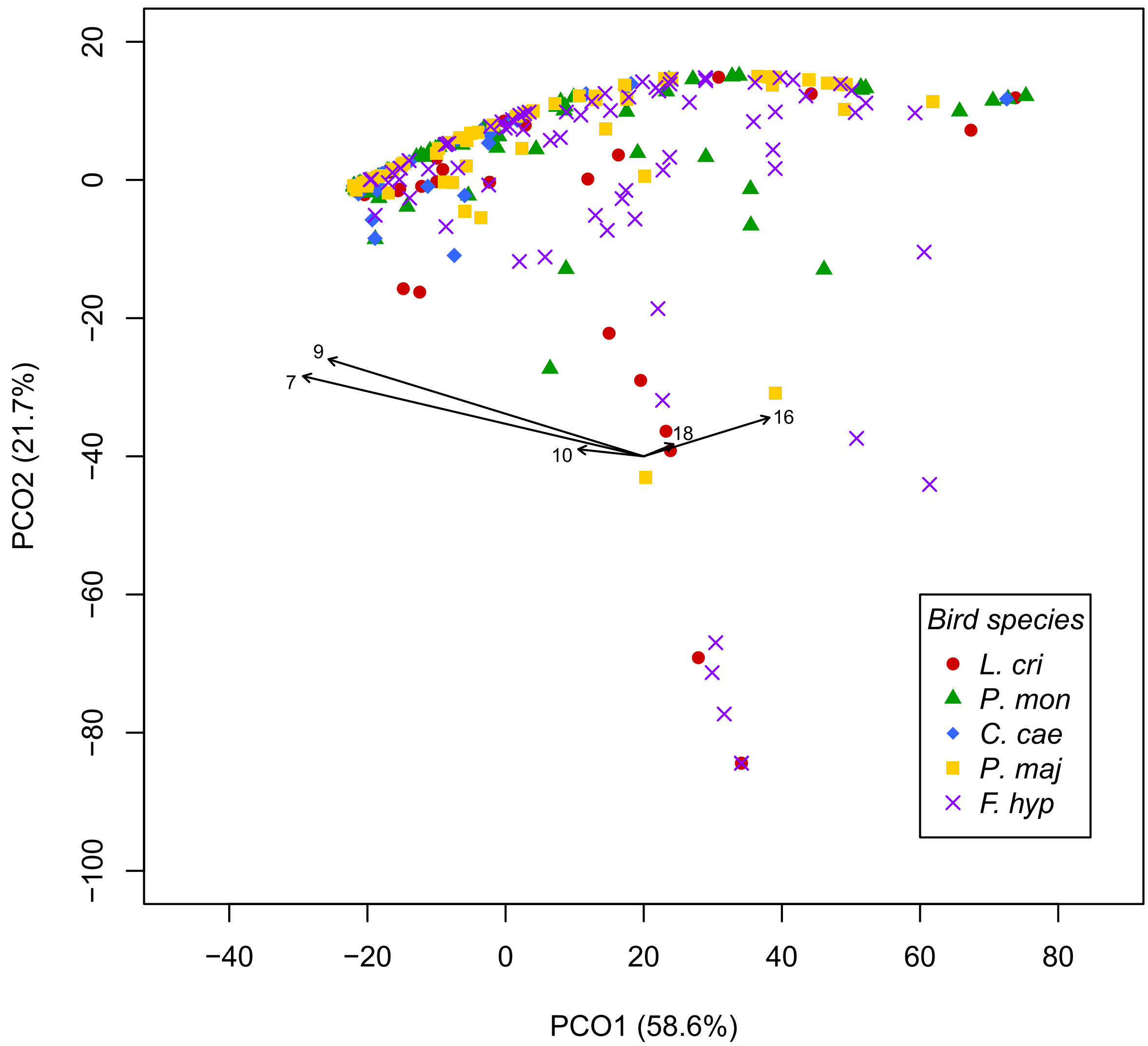

Figure S8. PCO ordinations of the bird species' diets analysed by prey order. Percentages next to the PCO axis titles refer to the percentage of variation explained by each. Vectors display Pearson correlations of prey traits and other variables, with relative lengths corresponding to their correlations to the axes. Only variables included in the best fitting DistLM models (lowest AICc or BIC, and scores within 1 of either) are shown (Table S7). Variables: 7 = Canopy, 9 = Deciduous, 10 = Coniferous, 16 = May date, 18= Latitude.

MOLECULAR ECOLOGY

Table S6. Best DistLM solutions (lowest AICc or BIC and scores within 1) for diets analysed by prey family. Variables: 7 = Canopy, 8 = Birch, 9 = Deciduous, 10 = Coniferous, 15 = Year, 16 = May date, 17 = Sequencing run, 18= Latitude.

Selected variables	AICc	BIC	R^2	Residual Sum of Squares	Number of variables
7–10, 16, 17	2623	2649.4	0.150	7.910E5	6
7, 9, 10, 16, 17	2623.5	2646.2	0.144	7.971E5	5
7–10, 16–18	2623.7	2653.8	0.154	7.876E5	7
7–10, 15–17	2623.8	2653.8	0.154	7.878E5	7
7, 9, 10, 16	2625.8	2644.7	0.133	8.074E5	4

Table S7. Best DistLM solutions (lowest AICc or BIC and scores within 1) for diets analysed by prey order. Variables: 7 = Canopy, 9 = Deciduous, 10 = Coniferous, 16 = May date, 18= Latitude.

Selected variables	AICc	BIC	R^2	Residual Sum of Squares	Number of variables
9, 10, 16	2257.0	2272.1	0.238	2.711E5	3
9, 10, 16, 18	2257.6	2276.5	0.242	2.699E5	4
7, 16	2264.3	2275.7	0.217	2.787E5	2

Table S8. Average similarities between and within bird species, following PERMANOVA with 999 permutations, S17 Bray–Curtis similarity, for diets analysed by prey family. Most differences were significant: crested tit × great tit t = 1.627, p = 0.018, all others t > 1.968, p ≤ 0.002.

	L. cri	*P. mon*	*C. cae*	*P. maj*	*F. hyp*
L. cri	20.001				
P. mon	23.693	33.267			
C. cae	28.656	38.376	46.264		
P. maj	23.394	30.150	35.324	29.602	
F. hyp	18.457	23.217	27.482	23.838	26.073

Table S9. Average similarities between and within bird species, following PERMANOVA with 999 permutations, S17 Bray–Curtis similarity, for diets analysed by prey order. Most differences were significant: willow tit × great tit t = 0.719, p = 0.592, crested tit × willow tit t = 1.823, p = 0.019, crested tit × great tit t = 2.082, p = 0.012, all others t ≥ 2.434, p ≤ 0.001.

	L. cri	*P. mon*	*C. cae*	*P. maj*	*F. hyp*
L. cri	47.885				
P. mon	55.984	65.813			
C. cae	62.723	74.115	87.668		
P. maj	57.374	67.604	74.482	69.475	
F. hyp	48.726	56.283	57.174	59.774	57.310

Table S10. Results of marginal tests for DistLM solutions for all prey species and sample variables for diets analysed by prey family.

Variable	SS (trace)	Pseudo-F	P-value	Proportion of variation explained
Canopy	53112	20.215	≤0.001	0.057
Birch	18278	6.691	≤0.001	0.020
Deciduous	42928	16.151	≤0.001	0.046
Coniferous	3894.6	1.404	0.151	0.004
Year	10913	3.963	0.003	0.012
May date	26769	9.891	≤0.001	0.029
Sequencing run	14143	5.154	≤0.001	0.015
Latitude	4669.6	1.684	0.095	0.005

Table S11. Results of marginal tests for DistLM solutions for all prey species and sample variables for diets analysed by prey order.

Variable	SS (trace)	Pseudo-F	P-value	Proportion of variation explained
Canopy	64189	73.501	≤0.001	0.180
Birch	11781	11.436	≤0.001	0.033
Deciduous	55835	62.155	≤0.001	0.157
Coniferous	2386.3	2.255	0.103	0.007
Year	2613.9	2.471	0.079	0.007
May date	11093	10.746	≤0.001	0.031
Sequencing run	2403.9	2.272	0.090	0.007
Latitude	1340.3	1.263	0.272	0.004

MOLECULAR ECOLOGY

Table S12. Results from models describing the effects of diet compositions on nestling weight in four bird species. Bold values indicate p<0.05. N(observations)=259; random effects included: Female identity (223), Year (3); df=23 for calculation of p-values. See Table S15 for further information on the random effects. "Pairwise" refers to relative effects for either bird species in the pair using the other as the baseline. Note that values for pairwise comparisons are listed only once for brevity; while technically slightly asymmetrical the numbers differed only for the third decimal place or less, with no impact on interpretation.

Diet	Fixed effect	β Estimate	Std. Error	t-value	p-value
Lepidopteran larvae	*L. cri*	11.978	0.211	56.877	**<0.001**
	P. mon	11.180	0.216	51.848	**<0.001**
	C. cae	9.259	0.423	21.912	**<0.001**
	P. maj	16.187	0.208	77.914	**<0.001**
	Female age adult	0.041	0.117	0.351	0.729
	Female age unknown	0.195	0.308	0.633	0.533
	L. cri × diet	−0.273	0.388	−0.705	0.488
	P. mon × diet	0.232	0.321	0.723	0.477
	C. cae × diet	1.579	0.514	3.072	**0.005**
	P. maj × diet	0.438	0.352	1.244	0.226
	pairwise *L. cri* & *P. mon*	0.505	0.499	1.013	0.322
	pairwise *L. cri* & *C. cae*	1.852	0.638	2.902	**0.008**
	pairwise *L. cri* & *P. maj*	0.711	0.524	1.356	0.188
	pairwise *P. mon* & *C. cae*	1.347	0.605	2.226	**0.036**
	pairwise *P. mon* & *P. maj*	0.206	0.471	0.437	0.666
	pairwise *C. cae* & *P. maj*	−1.141	0.624	−1.829	0.080
Lepidoptera	*L. cri*	11.656	0.320	36.456	**<0.001**
	P. mon	11.088	0.293	37.825	**<0.001**
	C. cae	8.985	0.742	12.111	**<0.001**
	P. maj	16.381	0.375	43.634	**<0.001**
	Female age adult	0.063	0.118	0.540	0.594
	Female age unknown	0.299	0.308	0.972	0.341
	L. cri × diet	0.378	0.412	0.919	0.368
	P. mon × diet	0.251	0.326	0.770	0.449
	C. cae × diet	1.635	0.789	2.072	**0.050**
	P. maj × diet	−0.039	0.466	−0.085	0.933
	pairwise *L. cri* & *P. mon*	−0.127	0.525	−0.242	0.811
	pairwise *L. cri* & *C. cae*	1.257	0.890	1.413	0.171
	pairwise *L. cri* & *P. maj*	−0.418	0.622	−0.672	0.508
	pairwise *P. mon* & *C. cae*	1.384	0.852	1.625	0.118
	pairwise *P. mon* & *P. maj*	−0.291	0.566	−0.514	0.612
	pairwise *C. cae* & *P. maj*	−1.675	0.914	−1.833	0.080

Levins' niche breadth	*L. cri*	12.033	0.279	43.149	**<0.001**
	P. mon	11.217	0.216	51.894	**<0.001**
	C. cae	10.667	0.295	36.207	**<0.001**
	P. maj	16.263	0.280	58.173	**<0.001**
	Female age adult	0.056	0.119	0.474	0.640
	Female age unknown	0.287	0.310	0.925	0.365
	L. cri × diet	−0.037	0.055	−0.670	0.510
	P. mon × diet	0.018	0.041	0.433	0.669
	C. cae × diet	−0.045	0.068	−0.666	0.512
	P. maj × diet	0.023	0.062	0.379	0.708
	pairwise *L. cri* & *P. mon*	0.055	0.069	0.798	0.433
	pairwise *L. cri* & *C. cae*	−0.008	0.087	−0.097	0.924
	pairwise *L. cri* & *P. maj*	0.060	0.082	0.733	0.471
	pairwise *P. mon* & *C. cae*	−0.063	0.080	−0.793	0.436
	pairwise *P. mon* & *P. maj*	0.005	0.074	0.074	0.942
	pairwise *C. cae* & *P. maj*	0.069	0.092	0.750	0.461

MOLECULAR ECOLOGY

Table S13. Results from models describing the effects of diet compositions on number of fledglings in five bird species. Bold values indicate $p<0.05$. N(observations)=336; random effects included: Year (3); df=321 used for calculation of p-values. See Table S15 for further information on the random effects. “Pairwise” refers to relative effects for either bird species in the pair using the other as the baseline. Note that values for pairwise comparisons are listed only once for brevity; while technically slightly asymmetrical the numbers differed only for the third decimal place or less, with no impact on interpretation.

Diet	Fixed effect	β Estimate	Std. Error	z-value	p-value
Lepidopteran larvae	*L. cri*	1.440	0.104	13.836	**<0.001**
	P. mon	1.844	0.090	20.575	**<0.001**
	C. cae	1.602	0.185	8.661	**<0.001**
	P. maj	1.748	0.091	19.286	**<0.001**
	F. hyp	1.713	0.155	11.085	**<0.001**
	Female age adult	0.108	0.051	2.105	**0.035**
	Female age unknown	0.043	0.123	0.347	0.729
	L. cri × diet	0.018	0.237	0.074	0.941
	P. mon × diet	0.077	0.138	0.553	0.580
	C. cae × diet	0.397	0.222	1.785	0.074
	P. maj × diet	0.162	0.165	0.981	0.326
	F. hyp × diet	0.032	0.175	0.181	0.856
	pairwise *L. cri* & *P. mon*	0.059	0.270	0.218	0.828
	pairwise *L. cri* & *C. cae*	0.379	0.324	1.170	0.242
	pairwise *L. cri* & *P. maj*	0.144	0.288	0.501	0.616
	pairwise *L. cri* & *F. hyp*	0.014	0.296	0.048	0.962
	pairwise *P. mon* & *C. cae*	0.320	0.260	1.231	0.218
	pairwise *P. mon* & *P. maj*	0.085	0.211	0.404	0.686
	pairwise *P. mon* & *F. hyp*	−0.045	0.227	−0.198	0.843
	pairwise *C. cae* & *P. maj*	−0.235	0.276	−0.852	0.394
	pairwise *C. cae* & *F. hyp*	−0.365	0.284	−1.287	0.198
	pairwise *P. maj* & *F. hyp*	−0.130	0.241	−0.540	0.589
Lepidoptera	*L. cri*	1.461	0.164	8.907	**<0.001**
	P. mon	1.830	0.132	13.877	**<0.001**
	C. cae	1.233	0.355	3.474	**<0.001**
	P. maj	1.715	0.157	10.917	**<0.001**
	F. hyp	1.675	0.165	10.181	**<0.001**
	Female age adult	0.118	0.051	2.289	**0.022**
	Female age unknown	0.063	0.121	0.522	0.602
	L. cri × diet	−0.038	0.217	−0.175	0.861
	P. mon × diet	0.060	0.153	0.389	0.697
	C. cae × diet	0.733	0.375	1.955	0.051
	P. maj × diet	0.124	0.193	0.641	0.522

	F. hyp × diet	0.060	0.183	0.326	0.744
	pairwise *L. cri* & *P. mon*	0.098	0.261	0.373	0.709
	pairwise *L. cri* & *C. cae*	0.771	0.430	1.793	0.073
	pairwise *L. cri* & *P. maj*	0.162	0.289	0.560	0.576
	pairwise *L. cri* & *F. hyp*	0.098	0.285	0.343	0.732
	pairwise *P. mon* & *C. cae*	0.674	0.400	1.686	0.092
	pairwise *P. mon* & *P. maj*	0.064	0.243	0.264	0.792
	pairwise *P. mon* & *F. hyp*	0.000	0.239	0.000	>0.999
	pairwise *C. cae* & *P. maj*	−0.609	0.419	−1.454	0.146
	pairwise *C. cae* & *F. hyp*	−0.673	0.418	−1.611	0.107
	pairwise *P. maj* & *F. hyp*	−0.064	0.266	−0.241	0.809
Levins' niche breadth	*L. cri*	1.539	0.127	12.136	**<0.001**
	P. mon	1.871	0.089	21.034	**<0.001**
	C. cae	1.863	0.118	15.802	**<0.001**
	P. maj	1.772	0.108	16.441	**<0.001**
	F. hyp	1.665	0.159	10.461	**<0.001**
	Female age adult	0.106	0.051	2.070	**0.039**
	Female age unknown	0.068	0.121	0.565	0.572
	L. cri × diet	−0.028	0.028	−0.998	0.318
	P. mon × diet	0.003	0.018	0.154	0.878
	C. cae × diet	0.014	0.028	0.512	0.609
	P. maj × diet	0.011	0.024	0.457	0.648
	F. hyp × diet	0.009	0.021	0.428	0.669
	pairwise *L. cri* & *P. mon*	0.031	0.033	0.928	**<0.001**
	pairwise *L. cri* & *C. cae*	0.043	0.040	1.072	**<0.001**
	pairwise *L. cri* & *P. maj*	0.039	0.037	1.061	**<0.001**
	pairwise *L. cri* & *F. hyp*	0.037	0.035	1.057	**<0.001**
	pairwise *P. mon* & *C. cae*	0.011	0.033	0.349	**<0.001**
	pairwise *P. mon* & *P. maj*	0.008	0.030	0.276	**<0.001**
	pairwise *P. mon* & *F. hyp*	0.006	0.027	0.228	**<0.001**
	pairwise *C. cae* & *P. maj*	−0.003	0.036	−0.091	**<0.001**
	pairwise *C. cae* & *F. hyp*	−0.005	0.034	−0.154	**<0.001**
	pairwise *P. maj* & *F. hyp*	−0.002	0.032	−0.062	**<0.001**

MOLECULAR ECOLOGY

Table S14. Results from models describing the effects of diet compositions on the probability to fledge in five bird species. Bold values indicate p<0.05. N(observations)=335; random effects included: Female identity (298), Year (3); df =22 for calculation of p-values. See Table S15 for further information on the random effects. "Pairwise" refers to relative effects for either bird species in the pair using the other as the baseline. Note that values for pairwise comparisons are listed only once for brevity; while technically slightly asymmetrical the numbers differed only for the third decimal place or less, with no impact on interpretation.

Diet	Fixed effect	*β* Estimate	Std. Error	z-value	p-value
Lepidopteran larvae	*L. cri*	3.354	0.513	6.537	**<0.001**
	P. mon	2.489	0.371	6.707	**<0.001**
	C. cae	−0.399	0.461	−0.864	0.388
	P. maj	1.106	0.290	3.813	**<0.001**
	F. hyp	4.404	0.719	6.126	**<0.001**
	Female age adult	0.041	0.170	0.241	0.809
	Female age unknown	−0.391	0.397	−0.986	0.324
	L. cri × diet	−0.515	1.043	−0.493	0.622
	P. mon × diet	0.781	0.602	1.297	0.195
	C. cae × diet	2.231	0.566	3.938	**<0.001**
	P. maj × diet	0.993	0.483	2.055	**0.040**
	F. hyp × diet	−1.323	0.935	−1.414	0.157
	pairwise *L. cri* & *P. mon*	1.295	1.198	1.081	0.280
	pairwise *L. cri* & *C. cae*	2.745	1.187	2.313	**0.021**
	pairwise *L. cri* & *P. maj*	1.508	1.149	1.313	0.189
	pairwise *L. cri* & *F. hyp*	−0.808	1.404	−0.575	0.565
	pairwise *P. mon* & *C. cae*	1.450	0.821	1.765	0.078
	pairwise *P. mon* & *P. maj*	0.212	0.760	0.279	0.780
	pairwise *P. mon* & *F. hyp*	−2.103	1.117	−1.883	0.060
	pairwise *C. cae* & *P. maj*	−1.238	0.738	−1.677	0.094
	pairwise *C. cae* & *F. hyp*	−3.553	1.092	−3.254	**0.001**
	pairwise *P. maj* & *F. hyp*	−2.316	1.050	−2.205	**0.027**
Lepidoptera	*L. cri*	3.104	0.747	4.154	**<0.001**
	P. mon	2.418	0.524	4.615	**<0.001**
	C. cae	−1.175	0.781	−1.503	0.133
	P. maj	0.781	0.460	1.698	0.090
	F. hyp	3.937	0.747	5.273	**<0.001**
	Female age adult	0.142	0.167	0.855	0.393
	Female age unknown	−0.204	0.392	−0.521	0.603
	L. cri × diet	0.064	1.017	0.063	0.950
	P. mon × diet	0.484	0.615	0.787	0.432
	C. cae × diet	2.680	0.834	3.215	**0.001**
	P. maj × diet	0.888	0.565	1.573	0.116

	F. hyp × diet	−0.723	0.956	−0.756	0.450
	pairwise *L. cri* & *P. mon*	0.420	1.184	0.354	0.723
	pairwise *L. cri* & *C. cae*	2.617	1.308	2.001	**0.045**
	pairwise *L. cri* & *P. maj*	0.824	1.161	0.710	0.478
	pairwise *L. cri* & *F. hyp*	−0.787	1.400	−0.562	0.574
	pairwise *P. mon* & *C. cae*	2.197	1.022	2.150	**0.032**
	pairwise *P. mon* & *P. maj*	0.405	0.823	0.492	0.623
	pairwise *P. mon* & *F. hyp*	−1.207	1.137	−1.061	0.289
	pairwise *C. cae* & *P. maj*	−1.792	0.993	−1.804	0.071
	pairwise *C. cae* & *F. hyp*	−3.403	1.272	−2.676	**0.007**
	pairwise *P. maj* & *F. hyp*	−1.611	1.110	−1.452	0.147
Levins' niche breadth	*L. cri*	3.467	0.639	5.428	**<0.001**
	P. mon	2.943	0.427	6.896	**<0.001**
	C. cae	1.912	0.415	4.608	**<0.001**
	P. maj	1.200	0.395	3.035	**0.002**
	F. hyp	3.615	0.698	5.178	**<0.001**
	Female age adult	0.124	0.171	0.725	0.469
	Female age unknown	−0.233	0.404	−0.578	0.563
	L. cri × diet	−0.067	0.124	−0.540	0.589
	P. mon × diet	−0.026	0.076	−0.345	0.730
	C. cae × diet	−0.159	0.083	−1.926	0.054
	P. maj × diet	0.070	0.079	0.879	0.379
	F. hyp × diet	0.002	0.109	0.020	0.984
	pairwise *L. cri* & *P. mon*	0.040	0.146	0.278	0.781
	pairwise *L. cri* & *C. cae*	−0.092	0.149	−0.620	0.536
	pairwise *L. cri* & *P. maj*	0.137	0.147	0.930	0.352
	pairwise *L. cri* & *F. hyp*	0.069	0.165	0.418	0.676
	pairwise *P. mon* & *C. cae*	−0.133	0.113	−1.175	0.240
	pairwise *P. mon* & *P. maj*	0.096	0.110	0.874	0.382
	pairwise *P. mon* & *F. hyp*	0.029	0.133	0.215	0.830
	pairwise *C. cae* & *P. maj*	0.229	0.114	2.006	0.045
	pairwise *C. cae* & *F. hyp*	0.162	0.136	1.191	0.234
	pairwise *P. maj* & *F. hyp*	−0.068	0.135	−0.502	0.616

MOLECULAR ECOLOGY

Table S15. Results for random effects for the models describing the effects of diet compositions on the birds' fitness. For nestling weight, N(observations)=259; Female identity (223), Year (3). For number of fledglings, N(observations)=336; Year (3). For probability to fledge, N(observations) = 335; Female identity (298), Year (3).

Fitness metric	Dietary metric	Variable					
		Female identity		Year		Residual	
		Variance	Std. Dev.	Variance	Std. Dev.	Variance	Std. Dev.
Nestling weight	Lepidopteran larvae	0.633	0.796	0.007	0.081	0.335	0.579
	Lepidoptera	0.600	0.774	0.003	0.056	0.382	0.618
	Levins' niche breadth	0.618	0.786	0.008	0.090	0.380	0.617
Number of fledglings	Lepidopteran larvae			0.001	0.030		
	Lepidoptera			0.001	0.029		
	Levins' niche breadth			0.001	0.035		
Probability to fledge	Lepidopteran larvae	0.528	0.727	0.055	0.234		
	Lepidoptera	0.535	0.731	0.044	0.209		
	Levins' niche breadth	0.625	0.791	0.108	0.328		
